# Australian guideline on management of diabetes-related foot infection: part of the 2021 Australian evidence-based guidelines for diabetes-related foot disease

**DOI:** 10.1186/s13047-022-00545-4

**Published:** 2022-06-09

**Authors:** Robert J. Commons, James Charles, Jane Cheney, Sarah A. Lynar, Matthew Malone, Edward Raby, on behalf of the Australian Diabetes-related Foot Disease Guidelines & Pathways Project

**Affiliations:** 1 Global Health Division, Menzies School of Health Research, Charles Darwin University, Darwin, Australia; 2 Internal Medical Services, Ballarat Health Services, Ballarat, Australia; 3 First Peoples Health Unit, Faculty of Health Griffith University, Gold Coast, Australia; 4 Diabetes Victoria, Melbourne, Australia; 5 Infectious Diseases Unit, Royal Darwin Hospital, Darwin, Australia; 6 South West Sydney Limb Preservation and Wound Research, South Western Sydney LHD, Liverpool, Sydney, Australia; 7 Infectious Diseases and Microbiology, School of Medicine Western Sydney University, Campbelltown Campus, Sydney, Australia; 8 Infectious Diseases Department, Royal Perth Hospital, Perth, Australia; 9 Department of Microbiology, Fiona Stanley Hospital Network, PathWest Laboratory Medicine, Murdoch, Australia; 10 Diabetes Feet Australia, Brisbane, Australia; 11 Australian Diabetes Society, Sydney, Australia

**Keywords:** Diabetes-related foot disease, Diabetes-related foot infection, Guidelines; antibiotic, Surgery

## Abstract

**Background:**

Diabetes-related foot infections cause substantial morbidity and mortality, both globally and in Australia. There is a need for up-to-date evidence-based guidelines to ensure optimal management of patients with diabetes-related foot infections. We aimed to identify and adapt high quality international guidelines to the Australian context to become the new Australian evidence-based guideline for people with a diabetes-related foot infection.

**Methods:**

Following Australian National Health and Medical Research Council (NHMRC) procedures we identified the 2019 International Working Group on the Diabetic Foot (IWGDF) guidelines as suitable for adaptation to the Australian context. Guidelines were screened, assessed and judged by an expert panel for the Australian context using the guideline adaptation frameworks ADAPTE and Grading of Recommendations Assessment, Development and Evaluation (GRADE). Judgements led to recommendations being adopted, adapted or excluded, with additional consideration regarding their implementation, monitoring and future research for the Australian context. Clinical pathways were then developed to assist implementation.

**Results:**

Of 36 original diabetes-related foot infection IWGDF sub-recommendations, 31 were adopted, four were adapted and one was excluded. Adaption was primarily undertaken due to differences or clarification of the sub-recommendations’ intended population. One sub-recommendation was excluded due to substantial differences in judgements between the panel and IWGDF and unacceptable heterogeneity of the target population. Therefore, we developed 35 evidence-based sub-recommendations for the Australian context that should guide best practice diagnosis and management of people with diabetes-related foot infection in Australia. Additionally, we incorporated these sub-recommendations into two clinical pathways to assist Australian health professionals to implement these evidence-based sub-recommendations into clinical practice. The six guidelines and the full protocol can be found at: https://www.diabetesfeetaustralia.org/new-guidelines/.

**Conclusions:**

A new national guideline for the diagnosis and management of people with diabetes-related foot infections were successfully developed for the Australian context. In combination with simplified clinical pathway tools they provide an evidence-based framework to ensure best management of individuals with diabetes-related foot infections across Australia and highlight considerations for implementation and monitoring.

**Supplementary Information:**

The online version contains supplementary material available at 10.1186/s13047-022-00545-4.

## Background

As the prevalence of diabetes mellitus continues to rise worldwide, there has been an increase in associated diabetes-related foot disease, including diabetes-related foot infections. Diabetes-related foot infections cause substantial morbidity and mortality, with an increasingly large economic impact, both directly through patient management and indirectly through patient disability [1]. Diabetes-related foot ulcers currently affect around 50,000 Australians [2], and up to 40% of these individuals can expect to have an associated infection in the first year after presentation [3].

Diabetes-related foot ulcers and infection are substantial risk factors for amputation, with 85% of all amputations in Australia associated with diabetes-related foot ulcers [4]. In Darwin, Australia, major amputations occurred in almost 10% of individuals with diabetes-related foot infections [5] presenting to hospital over 14 months and death in 9% over 1-year [6], with the median hospital stay lasting 29 days [5]. Risk of complications is further increased in Aboriginal and Torres Strait Islander Peoples [5, 7, 8] and addressing these risks is needed to successfully achieve key outcomes identified in the 2020 Closing the Gap in Partnership agreement [9].

A diabetes-related foot infection is defined as the presence of an infection in any tissue distal to the malleolus in an individual with diabetes mellitus. The majority of infections are associated with a breach of the epithelium (i.e. an ulcer). However, the presence of micro-organisms alone does not define the presence of an infection as a wound may be colonised by microorganisms. Thus, diagnosis generally requires the clinical recognition of inflammation [10]. Given the severe complications that can arise from diabetes-related foot infections, all infections, even those that are mild, should be considered serious.

Evidence-based guidelines are vital to ensure optimal multi-disciplinary management and outcomes of patients with diabetes-related foot infections. An Australian national evidence-based diabetes-related foot diseases guideline has not been published since 2011 [11] and is now out-of-date, having been rescinded by the National Health and Medical Research Council (NHMRC) [12]. New guidelines have been estimated to cost $AU1 million to develop [13], however, adopting or adapting suitable international guidelines can be an alternative efficient method of guideline development that is cost-effective. The current guideline aimed to identify and adapt high quality international guidelines to the Australian context to become the new Australian multi-disciplinary evidence-based guideline for people with a diabetes-related foot infection. This was undertaken in parallel with development of other Australian guidelines for people with other diabetes-related foot disease.

## Methods

The development of this guideline is described in detail in the accompanying guidelines development paper [14]. Guideline development followed Australian NHMRC recommendations for adapting international source guidelines [15, 16] combined with the ADAPTE process and GRADE Evidence to Decision (EtD) frameworks [17]. After defining the scope of the guidelines, international source guidelines were assessed with only the 2019 International Working Group of the Diabetic Foot (IWGDF) guidelines [10, 18–23] identified as suitable for adaptation. Recommendations relating to infection were identified [10]. Five further steps were then undertaken prior to the guideline presented here being finalised: i) assessing and deciding which source guideline recommendations to adopt, adapt or exclude in the new context; ii) drafting new recommendations and adding considerations for the Australian context; iii) collating recommendations and considerations into a new guideline; iv) developing clinical pathways to assist guideline implementation; and v) undertaking consultation and endorsement of the finalised guideline.

A six-member panel was convened to assess the 2019 IWGDF guidelines, including national experts in diabetes-related foot infection management and research together with consumer and Aboriginal and Torres Strait Islander experts. Each source sub-recommendation (and supporting IWGDF rationale and systematic review) was screened independently by two members of the panel for acceptability and applicability to the Australian context using a seven-item modified ADAPTE evaluation form [14, 16]. Disagreements were resolved through discussion, or by involving a third panel member if required. The entire panel then reviewed each sub-recommendation’s ratings to ensure consensus.

If the sub-recommendation was considered acceptable and applicable across all seven items, the sub-recommendation was adopted. If there were items where the panel was unsure or which were considered not acceptable or applicable the sub-recommendation underwent full assessment using a modified GRADE EtD framework consisting of eight criteria [14, 17, 24, 25]. One panel member populated the EtD template with all relevant evidence from the IWGDF guidelines and systematic reviews, and from relevant Australian literature or panel discussions during the ADAPTE process. Following review of the populated evidence by a second member, one member then rated the judgement items for each of the eight criteria. A second member then reviewed the judgements, with disagreements resolved through discussion, or a third panel member if required. The full panel then met and reviewed each of the summary judgements for the eight criteria to gain consensus and compared them to judgements made by the IWGDF.

The panel made a consensus decision to adopt, adapt or exclude the recommendation based on the degree of agreement between the panel and IWGDF judgements. Recommendations were adopted if no substantial disagreement existed between the panel and IWGDF judgement, were adapted if substantial disagreement existed or the population or intervention needed to be defined further, or were excluded if substantial disagreement existed and/or the panel considered the recommendation was not acceptable or applicable to the Australian setting. Consensus agreement was obtained through discussion, with review by the Guideline working group if needed.

The quality of evidence and strength of recommendation ratings and overall wording of recommendations that were adapted were reviewed by the panel based on the summary judgements of the GRADE EtD framework. The quality of evidence was rated according to GRADE methodology as high, moderate, low, or very low [24, 25]. The strength of recommendation was rated similarly according to GRADE methodology by weighing up the balance of effects in the Australian national context as strong or weak [24, 25].

All recommendations were collated into a draft manuscript including for each sub-recommendation a detailed rationale for the decision, summary justification for the judgements, detailed justification if the recommendation was fully assessed, and where applicable considerations for implementation, special subgroups (including for geographically remote, Aboriginal and Torres Strait Islander and other populations), monitoring and potential future research priorities in the Australian context. Finalised recommendations were then developed into infection diagnosis and management clinical pathways [14], to assist the implementation of these recommendations by health professionals in secondary and tertiary health care settings caring for Australians with diabetes-related foot infections. Pathways were developed using a 10-step process as described by Flores et al. [14, 26].

A six-week public consultation period was undertaken from March 2021 using a customised consultation survey from ADAPTE [14, 16]. The manuscript was revised following collation and review of the survey feedback. Endorsement was then sought from the Guidelines Development working group, and the Australasian Society of Infectious Diseases prior to release of the final guideline [14] in conjunction with five other individual sub-field guidelines [27–31].

## Results

All 27 recommendations, including 36 separate sub-recommendations, were systematically evaluated. After screening, 29 sub-recommendations were adopted and seven required full assessment (Table 1). Of the seven sub-recommendations assessed, two were adopted without change, four were adapted, and one was excluded (Table 2). Of the four that were adapted, one had the strength of recommendation changed, one had the quality of evidence changed and four had the population adjusted or clarified. The sub-recommendation that was excluded was considered to have too heterogeneous a population and be covered by alternative recommendations. Wording differences between IWGDF and Australian guidelines are summarised in Table 3.

**Table 1:** Summary of screening ratings for acceptability and applicability in the Australian context for all IWGDF Infection recommendations

Recommendation	Acceptability			Applicability				Full assessment	Comments
	1	2	3	4	5	6	7		
1a	+	+	+	+	+	+	+	No	
1b	+	+	+	+	+	+	+	No	
2	+	+	+	+	+	+	+	No	
3	+	+	+	+	+	+	+	No	
4	+	+	+	+	+	+	+	No	
5	+	+	+	+	+	+	+	No	
6a	+	+	+	+	+	+	+	No	
6b	+	+	+	+	+	+	+	No	
7	+	+	+	+	+	+	+	No	
8a	+	+	+	+	+	+	+	No	
8b	+	+	+	+	+	+	+	No	
9	+	+	+	+	+	+	+	No	
10	+	+	+	+	+	+	+	No	
11	+	+	+	+	+	+	+	No	
12	+	+	+	–	+	+	+	Yes	Assess applicability to patient population
13	+	+	+	+	+	+	+	No	
14	+	+	+	+	+	+	+	No	
15a	+	+	+	+	+	+	+	No	
15b	+	+	+	+	+	+	+	No	
15c	+	+	+	+	+	+	+	No	
16	+	+	+	?	+	+	+	Yes	Assess applicability to patient population
17	+	+	+	?	+	+	+	Yes	Assess applicability to patient population
18	+	+	+	?	+	+	+	Yes	Assess applicability to patient population
19	+	+	+	+	+	+	+	No	
20	+	+	+	+	+	+	+	No	
21a	+	+	+	?	+	+	+	Yes	Assess applicability to patient population
21b	+	+	+	+	+	+	+	No	
22	+	+	+	+	+	+	+	No	
23a	+	+	+	+	+	–	–	Yes	Assess expertise availability and policy constraints
23b	+	+	+	+	+	+	+	No	
24	+	+	+	+	+	+	+	No	
25a	+	+	+	+	+	+	+	No	
25b	+	?	+	+	+	+	+	Yes	Assess strength of recommendation
26	+	+	+	+	+	+	+	No	
27a	+	+	+	+	+	+	+	No	
27b	+	+	+	+	+	+	+	No	
Total	**36**	**35**	**36**	**31**	**36**	**35**	**35**	**7**	
%	**100**	**97**	**100**	**86**	**100**	**97**	**97**	**19**	

*Note*: +, yes item is met; −, no item is not met;? unsure if item is met

**Table 2:** Summary of final panel judgements compared with IWGDF judgements for all IWGDF Infection recommendations

No	Problem	Desirable effects	Undesirable effects	Quality of evidence	Values	Balance of effects	Acceptability	Applicability/feasibility	Decision	Comment
1a	=	=	=	=	=	=	=	=	Adopt	Adopted in screening
1b	=	=	=	=	=	=	=	=	Adopt	Adopted in screening
2	=	=	=	=	=	=	=	=	Adopt	Adopted in screening
3	=	=	=	=	=	=	=	=	Adopt	Adopted in screening
4	=	=	=	=	=	=	=	=	Adopt	Adopted in screening
5	=	=	=	=	=	=	=	=	Adopt	Adopted in screening
6a	=	=	=	=	=	=	=	=	Adopt	Adopted in screening
6b	=	=	=	=	=	=	=	=	Adopt	Adopted in screening
7	=	=	=	=	=	=	=	=	Adopt	Adopted in screening
8a	=	=	=	=	=	=	=	=	Adopt	Adopted in screening
8b	=	=	=	=	=	=	=	=	Adopt	Adopted in screening
9	=	=	=	=	=	=	=	=	Adopt	Adopted in screening
10	=	=	=	=	=	=	=	=	Adopt	Adopted in screening
11	=	=	=	=	=	=	=	=	Adopt	Adopted in screening
12	Probably yes	Moderate	Trivial	Very low	Possibly important uncertainty	Probably favours the intervention	Probably yes	Yes	Adapt	Adapted QoE & population
13	=	=	=	=	=	=	=	=	Adopt	Adopted in screening
14	=	=	=	=	=	=	=	=	Adopt	Adopted in screening
15a	=	=	=	=	=	=	=	=	Adopt	Adopted in screening
15b	=	=	=	=	=	=	=	=	Adopt	Adopted in screening
15c	=	=	=	=	=	=	=	=	Adopt	Adopted in screening
16	Probably yes	Small	Trivial	Low	Possibly important uncertainty	Probably favours the intervention	Probably yes	Probably yes	Adapt	Adapted strength of recommendation and population
17	Probably yes	Moderate	Trivial	Low	Possibly important uncertainty	Probably favours the intervention	Probably yes	Probably yes	Adapt	Adapted population
18	Probably yes	Small	Trivial	Low	Possibly important uncertainty	Probably favours the intervention	Probably yes	Probably yes	Adapt	Adapted population
19	=	=	=	=	=	=	=	=	Adopt	Adopted in screening
20	=	=	=	=	=	=	=	=	Adopt	Adopted in screening
21a	Probably yes	Moderate	Trivial	Moderate	Possibly important uncertainty	Favours the intervention	Probably yes	Probably yes	Adopt	Adopted with full assessment
21b	=	=	=	=	=	=	=	=	Adopt	Adopted in screening
22	=	=	=	=	=	=	=	=	Adopt	Adopted in screening
23a	Probably yes	Trivial	Varies	Low	Probably no important uncertainty	Varies	Varies	Probably yes	Exclude	Excluded due to balance of effects, quality of evidence and population
23b	=	=	=	=	=	=	=	=	Adopt	Adopted in screening
24	=	=	=	=	=	=	=	=	Adopt	Adopted in screening
25a	=	=	=	=	=	=	=	=	Adopt	Adopted in screening
25b	Probably yes	Small	Trivial	Moderate	Possibly important uncertainty	Probably favours the intervention	Probably yes	Probably yes	Adopt	Adopted with full assessment
26	=	=	=	=	=	=	=	=	Adopt	Adopted in screening
27a	=	=	=	=	=	=	=	=	Adopt	Adopted in screening
27b	=	=	=	=	=	=	=	=	Adopt	Adopted in screening

*Note*: +, panel agreed with original IWGDF judgement; −, panel disagreed with original IWGDF judgement;?, panel unsure if agreed with original IWGDF judgement due to lack of IWGDF information on judgement; =, panel agreed with original IWGDF judgements during screening (see Table 1); QoE: Quality of evidence

**Table 3:** Summary of the original IWGDF recommendation compared with the new Australian guideline recommendations for diabetes-related foot infections

No	Original IWGDF Recommendation	Decision	New Australian Recommendation
1a	Diagnose a soft tissue diabetic foot infection clinically, based on the presence of local or systemic signs and symptoms of inflammation. (Strong; low)	Adopted	Diagnose a soft tissue diabetes-related foot infection clinically, based on the presence of local or systemic signs and symptoms of inflammation. (Strong; low)
1b	Assess the severity of any diabetic foot infection using the Infectious Diseases Society of America/International Working Group on the Diabetic Foot classification scheme. (Strong; moderate)	Adopted	Assess the severity of any diabetes-related foot infection using the International Working Group on the Diabetic Foot / Infectious Diseases Society of America classification scheme. (Strong; moderate)
2	Consider hospitalising all persons with diabetes and a severe (grade 4) foot infection and those with a moderate (grade 3) infection that is complex or associated with key relevant morbidities. (Strong; low)	Adopted	As stated in original IWGDF Recommendation
3	In a person with diabetes and a possible foot infection for whom the clinical examination is equivocal or uninterpretable, consider ordering an inflammatory serum biomarker, such as C-reactive protein, erythrocyte sedimentation rate, and perhaps procalcitonin, as an adjunctive measure for establishing the diagnosis. (Weak; low)	Adopted	As stated in original IWGDF Recommendation
4	As neither electronically measuring foot temperature nor using quantitative microbial analysis has been demonstrated to be useful as a method for diagnosing diabetic foot infection, we suggest not using them. (Weak; low)	Adopted	As neither electronically measuring foot temperature nor using quantitative microbial analysis has been demonstrated to be useful as a method for diagnosing diabetes-related foot infection, we suggest not using them. (Weak; low)
5	In a person with diabetes and suspected osteomyelitis of the foot, we recommend using a combination of the probe-to-bone test, the erythrocyte sedimentation rate (or C-reactive protein and/or procalcitonin), and plain X-rays as the initial studies to diagnose osteomyelitis. (Strong; moderate)	Adopted	As stated in original IWGDF Recommendation
6a	In a person with diabetes and suspected osteomyelitis of the foot, if a plain X-ray and clinical and laboratory findings are most compatible with osteomyelitis, we recommend no further imaging of the foot to establish the diagnosis. (Strong; low)	Adopted	As stated in original IWGDF Recommendation
6b	If the diagnosis of osteomyelitis remains in doubt, consider ordering an advanced imaging study, such as magnetic resonance imaging scan, 18F-FDG-positron emission tomography/computed tomography (CT) or leukocyte scintigraphy (with or without CT). (Strong; moderate)	Adopted	If the diagnosis of osteomyelitis remains in doubt, consider ordering an advanced imaging study, such as magnetic resonance imaging scan, 18F-FDG-positron emission tomography (PET)/computed tomography (CT) or leukocyte scintigraphy (with or without CT). (Strong; moderate)
7	In a person with diabetes and suspected osteomyelitis of the foot, in whom making a definitive diagnosis or determining the causative pathogen is necessary for selecting treatment, collect a sample of bone (percutaneously or surgically) to culture clinically relevant bone microorganisms and for histopathology (if possible). (Strong; low)	Adopted	As stated in original IWGDF Recommendation
8a	Collect an appropriate specimen for culture for almost all clinically infected wounds to determine the causative pathogens. (Strong; low)	Adopted	As stated in original IWGDF Recommendation
8b	For a soft tissue diabetic foot infection, obtain a sample for culture by aseptically collecting a tissue specimen (by curettage or biopsy) from the ulcer. (Strong; moderate)	Adopted	For a soft tissue diabetes-related foot infection, obtain a sample for culture by aseptically collecting a tissue specimen (by curettage or biopsy) from the ulcer. (Strong; moderate)
9	Do not use molecular microbiology techniques (instead of conventional culture) for the first-line identification of pathogens from samples in a patient with a diabetic foot infection. (Strong; low)	Adopted	Do not use molecular microbiology techniques (instead of conventional culture) for the first-line identification of pathogens from samples in a patient with a diabetes-related foot infection. (Strong; low)
10	Treat a person with a diabetic foot infection with an antibiotic agent that has been shown to be effective in a published randomized controlled trial and is appropriate for the individual patient. Some agents to consider include penicillins, cephalosporins, carbapenems, metronidazole (in combination with other antibiotic [s]), clindamycin, linezolid, daptomycin, fluoroquinolones, or vancomycin, but not tigecycline. (Strong; high)	Adopted	Treat a person with a diabetes-related foot infection with an antibiotic agent that has been shown to be effective in a published randomised controlled trial and is appropriate for the individual patient. Some agents to consider include penicillins, cephalosporins, carbapenems, metronidazole (in combination with other antibiotic [s]), clindamycin, linezolid, daptomycin, fluoroquinolones, or vancomycin, but not tigecycline. (Strong; high)
11	Select an antibiotic agent for treating a diabetic foot infection based on: the likely or proven causative pathogen(s) and their antibiotic susceptibilities; the clinical severity of the infection; published evidence of efficacy of the agent for diabetic foot infections; risk of adverse events, including collateral damage to the commensal flora; likelihood of drug interactions; agent availability; and, financial costs. (Strong; moderate)	Adopted	Select an antibiotic agent for treating a diabetes-related foot infection based on: the likely or proven causative pathogen(s) and their antibiotic susceptibilities; the clinical severity of the infection; published evidence of efficacy of the agent for diabetes-related foot infections; risk of adverse events, including collateral damage to the commensal flora; likelihood of drug interactions; agent availability; and, financial costs. (Strong; moderate)
12	Administer antibiotic therapy initially by the parenteral route to any patient with a severe (grade 4) diabetic foot infection. Switch to oral therapy if the patient is clinically improving and has no contraindications to oral therapy and if there is an appropriate oral agent available. (Strong; low)	Adapted	Administer antibiotic therapy initially by the parenteral route to any patient with a severe (grade 4) skin and soft tissue diabetes-related foot infection. Switch to oral therapy if the patient is clinically improving and has no contraindications to oral therapy and if there is an appropriate oral agent available. (Strong; very low)
13	Treat patients with a mild (grade 2) diabetic foot infection, and most with a moderate (grade 3) diabetic foot infection, with oral antibiotic therapy, either at presentation or when clearly improving with initial intravenous therapy. (Weak; low)	Adopted	Treat patients with a mild (grade 2) diabetes-related foot infection, and most with a moderate (grade 3) diabetes-related foot infection, with oral antibiotic therapy, either at presentation or when clearly improving with initial intravenous therapy. (Weak; low)
14	We suggest not using any currently available topical antimicrobial agent for treating a mild (grade 2) diabetic foot infection. (Weak; moderate)	Adopted	We suggest not using any currently available topical antimicrobial agent for treating a mild (grade 2) diabetes-related foot infection. (Weak; moderate)
15a	Administer antibiotic therapy to a patient with a skin or soft tissue diabetic foot infection for a duration of 1 to 2 weeks. (Strong; high)	Adopted	Administer antibiotic therapy to a patient with a skin or soft tissue diabetes-related foot infection for a duration of 1 to 2 weeks. (Strong; high)
15b	Consider continuing treatment, perhaps for up to 3 to 4 weeks, if the infection is improving but is extensive and is resolving slower than expected or if the patient has severe peripheral artery disease. (Weak; low)	Adopted	As stated in original IWGDF Recommendation
15c	If evidence of infection has not resolved after 4 weeks of apparently appropriate therapy, re-evaluate the patient, and reconsider the need for further diagnostic studies or alternative treatments. (Strong; low)	Adopted	As stated in original IWGDF Recommendation
16	For patients who have not recently received antibiotic therapy and who reside in a temperate climate area, target empiric antibiotic therapy at just aerobic gram-positive pathogens (beta-haemolytic streptococci and *S. aureus*) in cases of a mild (grade 2) diabetic foot infection. (Strong; low)	Adapted	For patients who have not recently received antibiotic therapy and have an acute infection, consider targeting empiric antibiotic therapy at just aerobic Gram positive pathogens (beta-haemolytic streptococci and *Staphylococcus* *aureus*) in cases of a mild (grade 2) diabetes-related foot infection. (Weak; low)
17	For patients residing in a tropical/subtropical climate, or who have been treated with antibiotic therapy within a few weeks, have a severely ischemic affected limb, or a moderate (grade 3) or severe (grade 4) infection, we suggest selecting an empiric antibiotic regimen that covers gram positive pathogens, commonly isolated gram-negative pathogens, and possibly obligate anaerobes in cases of moderate (grade 3) to severe (grade 4) diabetic foot infections. Then, reconsider the antibiotic regimen based on both the clinical response and culture and sensitivity results. (Weak; low)	Adapted	For patients who have been treated with antibiotic therapy within a few weeks, have a chronic infection, have a severely ischaemic affected limb, or a moderate (grade 3) or severe (grade 4) infection, we suggest selecting an empiric antibiotic regimen that covers Gram positive pathogens, commonly isolated Gram negative pathogens, and possibly obligate anaerobes in cases of moderate (grade 3) to severe (grade 4) diabetes-related foot infections. Then, reconsider the antibiotic regimen based on both the clinical response and culture and sensitivity results. (Weak; low)
18	Empiric treatment aimed at *Pseudomonas aeruginosa* is not usually necessary in temperate climates, but consider it if *P. aeruginosa* has been isolated from cultures of the affected site within the previous few weeks, or in tropical/subtropical climates (at least for moderate (grade 3) or severe (grade 4) infection). (Weak; low)	Adapted	Empiric treatment aimed at *Pseudomonas aeruginosa* is not usually necessary but consider it if *P. aeruginosa* has been isolated from cultures of the affected site within the previous few weeks, or in tropical/subtropical climates (at least for moderate [grade 3] or severe [grade 4] infection). (Weak; low)
19	Do not treat clinically uninfected foot ulcers with systemic or local antibiotic therapy with the goal of reducing the risk of infection or promoting ulcer healing. (Strong; low)	Adopted	As stated in original IWGDF Recommendation
20	Nonsurgeons should urgently consult with a surgical specialist in cases of severe (grade 4) infection or of moderate (grade 3) infection complicated by extensive gangrene, necrotizing infection, signs suggesting deep (below the fascia) abscess or compartment syndrome, or severe lower limb ischemia. (Strong; low)	Adopted	Nonsurgeons should urgently consult with a surgical specialist in cases of severe (grade 4) infection or of moderate (grade 3) infection complicated by extensive gangrene, necrotising infection, signs suggesting deep (below the fascia) abscess or compartment syndrome, or severe lower limb ischaemia. (Strong; low)
21a	In a patient with diabetes and uncomplicated forefoot osteomyelitis, for whom there is no other indication for surgical treatment, consider treating with antibiotic therapy without surgical resection of bone. (Strong; moderate)	Adopted	As stated in original IWGDF Recommendation
21b	In a patient with probable diabetic foot osteomyelitis with concomitant soft tissue infection, urgently evaluate for the need for surgery as well as intensive post-operative medical and surgical follow-up. (Strong; moderate)	Adopted	In a patient with probable diabetes-related foot osteomyelitis with concomitant soft tissue infection, urgently evaluate the need for surgery as well as intensive post-operative medical and surgical follow-up. (Strong; moderate)
22	Select antibiotic agents for treating diabetic foot osteomyelitis from among those that have demonstrated efficacy for osteomyelitis in clinical studies. (Strong; low)	Adopted	Select antibiotic agents for treating diabetes-related foot osteomyelitis from among those that have demonstrated efficacy for osteomyelitis in clinical studies. (Strong; low)
23a	Treat diabetic foot osteomyelitis with antibiotic therapy for no longer than 6 weeks. If the infection does not clinically improve within the first 2 to 4 weeks, reconsider the need for collecting a bone specimen for culture, undertaking surgical resection, or selecting an alternative antibiotic regimen. (Strong; moderate)	Excluded	
23b	Treat diabetic foot osteomyelitis with antibiotic therapy for just a few days if there is no soft tissue infection and all the infected bone has been surgically removed. (Weak; low)	Adopted	Treat diabetes-related foot osteomyelitis with antibiotic therapy for just a few days if there is no soft tissue infection and all the infected bone has been surgically removed. (Weak; low)
24	For diabetic foot osteomyelitis cases that initially require parenteral therapy, consider switching to an oral antibiotic regimen that has high bioavailability after perhaps 5 to 7 days, if the likely or proven pathogens are susceptible to an available oral agent and the patient has no clinical condition precluding oral therapy. (Weak; moderate)	Adopted	For people with diabetes-related foot osteomyelitis that initially require parenteral therapy, consider switching to an oral antibiotic regimen that has high bioavailability after perhaps 5 to 7 days, if the likely or proven pathogens are susceptible to an available oral agent and the patient has no clinical condition precluding oral therapy. (Weak; moderate)
25a	During surgery to resect bone for diabetic foot osteomyelitis, consider obtaining a specimen of bone for culture (and, if possible, histopathology) at the stump of the resected bone to identify if there is residual bone infection. (Weak; moderate)	Adopted	During surgery to resect bone for diabetes-related foot osteomyelitis, consider obtaining a specimen of bone for culture (and, if possible, histopathology) at the stump of the resected bone to identify if there is residual bone infection. (Weak; moderate)
25b	If an aseptically collected culture specimen obtained during the surgery grows pathogen(s), or if the histology demonstrates osteomyelitis, administer appropriate antibiotic therapy for up to 6 weeks. (Strong; moderate)	Adopted	As stated in original IWGDF Recommendation
26	For a diabetic foot infection, do not use hyperbaric oxygen therapy or topical oxygen therapy as an adjunctive treatment if the only indication is specifically for treating the infection. (Weak; low)	Adopted	For a diabetes-related foot infection, do not use hyperbaric oxygen therapy or topical oxygen therapy as an adjunctive treatment if the only indication is specifically for treating the infection. (Weak; low)
27a	To specifically address infection in a diabetic foot ulcer do not use adjunctive granulocyte colony stimulating factor treatment (Weak; moderate)	Adopted	As stated in original IWGDF Recommendation
27b	To specifically address infection in a diabetic foot ulcer do not routinely use topical antiseptics, silver preparations, honey, bacteriophage therapy, or negative pressure wound therapy (with or without instillation). (Weak; low)	Adopted	To *specifically* address infection in a diabetes-related foot ulcer do not *routinely* use topical antiseptics, silver preparations, honey, bacteriophage therapy, or negative pressure wound therapy (with or without instillation). (Weak; low)

*Note*: underlined wording indicates the specific adapted changes to the original IWGDF recommendation

Four responses were received to the public consultation survey with three responding that they agreed that the guideline should be approved as the new Australian Infection guideline, that the guideline would be supported by the majority of their colleagues and all agreed if approved they would encourage its use in practice. All de-identified feedback comments received during public consultation and the panel’s responses to each comment were collated and posted on the Diabetes Feet Australia website. Based on the collated public consultation feedback, the guideline was revised, approved by the panel and Australian DFD Guidelines working group, and endorsed as the new *Australian guideline on management of diabetes-related foot infection* by nine peak national bodies including the Australian and New Zealand Society for Vascular Surgery, Australian Podiatry Association, Wounds Australia, Australasian Society for Infectious Diseases, Australian Orthotic Prosthetic Association, Pedorthic Association of Australia, Australian Aboriginal and Torres Strait Islander Diabetes-related Foot Complications Program, Australian Diabetes Society and Diabetes Feet Australia.

**Fig. 1:**
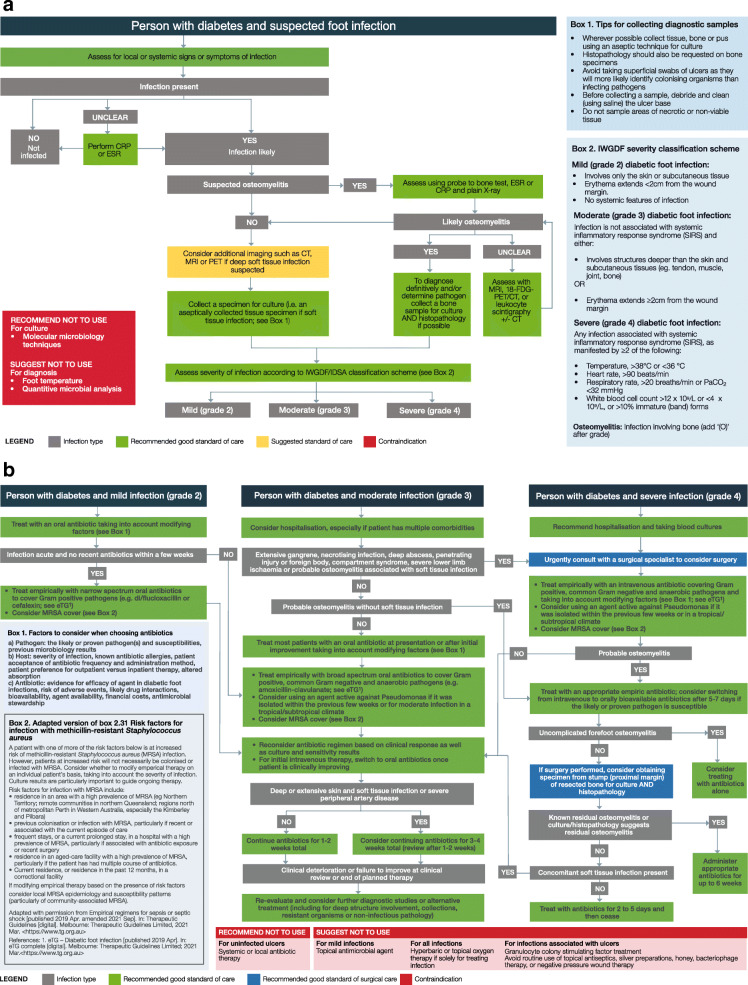
**a** Australian clinical pathway to guide evidence-based diagnosis of infection in people with diabetes. **b** Australian clinical pathway to guide evidence-based management of infection in people with diabetes

Figure 1a and b incorporate the updated Australian recommendations in two clinical pathways to guide evidence-based diagnosis and treatment of people with diabetes-related foot infection in Australia. For each of the sub-recommendations, we outline below: the population, intervention, control and outcome (PICO) framed question the recommendation addressed in the IWGDF guidelines; the Australian recommendation; the panel decision and rationale to adopt, adapt or exclude; summary justification for the recommendation; detailed justification if it underwent full assessment; and where identified, considerations for implementation, special subgroups (including for Aboriginal and Torres Strait Islander and geographically remote populations), monitoring and potential future research priorities.

## Question one part a

In a person with diabetes and a foot infection, do increasing levels of severity of the IWGDF/Infectious Diseases Society of America (IDSA) criteria correlate with increasing rates of adverse outcomes (e.g., need for hospitalisation, failure to resolve infection, or lower extremity amputation)?

### Recommendation 1a

Diagnose a soft tissue diabetes-related foot infection clinically, based on the presence of local or systemic signs and symptoms of inflammation. (GRADE strength of recommendation: Strong; Quality of evidence: low).

#### Decision: Adopted

Rationale: The panel decided to adopt the recommendation unchanged following screening, as judgements were consistent with the IWGDF and the recommendation was acceptable and applicable in the Australian setting (Table 1).

#### Summary justification

The panel agreed with the IWGDF that despite a low quality of evidence for the recommendation, there was a strong (strength of) recommendation for it based on the clinical signs and symptoms of infection being widely accepted and the substantial benefits of rapid clinical identification of soft tissue diabetes-related foot infections. The recommendation was considered compatible and applicable to the Australian context, and the clinical expertise was considered to be available for its implementation across primary, secondary and tertiary healthcare providers.

#### Subgroup considerations

##### Aboriginal and Torres Strait islander peoples

Clinical diagnosis of soft tissue infections may be more difficult in individuals with dark skin due to decreased contrast between infected and non-infected skin. This may be more likely for clinicians that treat individuals with dark skin less commonly.

##### Other subgroup considerations

As with Aboriginal and Torres Strait Islander Peoples, clinical diagnosis may be more difficult in other groups with darker skin.

#### Future research considerations

The panel identified that future studies could investigate differences in the time to identify an infected foot in individuals with darker and lighter skin and could define differences in clinical symptoms of infection between these two groups in order to adapt tools that use these clinical symptoms of infection to individuals with different skin colours.

### Recommendation 1b

Assess the severity of any diabetes-related foot infection using the International Working Group on the Diabetic Foot / Infectious Diseases Society of America classification scheme. (Strong; moderate).

#### Decision: Adopted

Rationale: The panel decided to adopt the recommendation unchanged following screening as judgements were consistent with the IWGDF and the recommendation was considered acceptable and applicable in the Australian setting (Table 1).

#### Summary justification

The panel agreed with the IWGDF that there was a moderate quality of evidence and a strong (strength of) recommendation for the recommendation given that the classification system has been validated in full or part by three prospective and four retrospective cohort studies. The recommendation was considered compatible and applicable to the Australian context, and the panel considered that the clinical expertise for its implementation was available across primary, secondary and tertiary healthcare providers.

#### Implementation considerations

The panel noted that there are a number of versions of the IWGDF/IDSA classification scheme with the IWGDF classification scheme being the most recently updated in 2019 as part of the IWGDF guideline recommendation process. As such, this classification scheme is recommended for use in Australia. In addition, the panel noted that a number of clinicians working with patients with diabetes-related foot infections use the Society for Vascular Surgery Wound, Ischaemia and Foot Infection (WIfI) classification scheme [32] to assess diabetes-related foot disease. This includes an infection component which is very similar to the IWGDF classification scheme although it ranges from 0 (no infection) to 3 (severe infection) compared with 1 (no infection) to 4 (severe infection in the IWGDF classification scheme. In addition, it does not include the changes suggested by the 2019 IWGDF Guidelines to incorporate an ‘O’ when patients with moderate or severe infection have associated osteomyelitis.

### Subgroup considerations

#### Geographically remote people

The panel noted that some components of the classification scheme associated with identifying systemic inflammatory response syndrome (SIRS) such as measurement of the PaCO_2_, white blood cell count or band forms may not be available in all centres, however, inability to assess these was likely to only marginally impact on the sensitivity of identifying SIRS.

#### Monitoring considerations

The panel suggests that services use the IWGDF classification scheme to categorise patients in their service to assist with subsequent planning and management decisions.

#### Future research considerations

The panel noted that given the ongoing changes to the IWGDF guidelines and no studies having validated these in the Australian context it would be beneficial to conduct further validation studies of this classification scheme in the Australian setting.

## Question one part b

Which persons presenting with diabetes and foot infection should be hospitalised for management of infection?

### Recommendation 2

Consider hospitalising all persons with diabetes and a severe (grade 4) foot infection and those with a moderate (grade 3) infection that is complex or associated with key relevant morbidities. (Strong; low).

#### Decision: Adopted

Rationale: The panel decided to adopt the recommendation unchanged following screening as judgements were consistent with the IWGDF and the recommendation was considered acceptable and applicable in the Australian setting (Table 1).

#### Summary justification

The panel agreed with the IWGDF that there was a low quality of evidence but a strong (strength of) recommendation for the recommendation given that the desirable effects of hospitalisation on infection resolution, wound healing and mortality prevention in this setting likely outweigh the undesirable effects. The recommendation was considered compatible with most patients’ values, applicable to the Australian context and feasible in most Australian locations, although they noted that some patient subgroups may disagree and in some remote locations the application of this recommendation would be more difficult.

#### Subgroup considerations

##### Geographically remote people

The panel noted there is a disparity across Australia in regard to access to multidisciplinary teams (MDT)-High Risk Foot Services or centres with specialist surgical services to manage patients with some moderate (grade 3) and severe (grade 4) diabetes-related foot infections requiring urgent or elective surgical intervention. This is particularly pertinent for individuals in remote areas where the adoption of more out-patient based managements may be preferred such as chairside surgeries or the utilisation of out-patient parenteral antibiotic therapy.

##### Aboriginal and Torres Strait islander peoples

Similar to people in geographically remote locations it was noted that some Aboriginal and Torres Strait Islander Peoples may be located in remote areas where hospitalisation is associated with substantial barriers, and alternative treatments may be considered preferable in some circumstances. If hospitalisation is required, the panel highlighted the need for adequate consultation with the patient and engagement with family to explain why hospitalisation is required and the approximate length of stay. There should also be consideration of language barriers and the need for a professional interpreter, especially where English may be a second, third or fourth language.

#### Future research considerations

The panel noted that there is a need to compare outcomes from alternative models of practice that may be of particular relevance to remote locations and Aboriginal and Torres Strait Islander communities around Australia.

## Question two part a

In a person with diabetes and a suspected foot infection, how well do the IWGDF/IDSA clinical criteria for diagnosing soft tissue infection correlate with other diagnostic tests?

### Recommendation 3

In a person with diabetes and a possible foot infection for whom the clinical examination is equivocal or uninterpretable, consider ordering an inflammatory serum biomarker, such as C-reactive protein (CRP), erythrocyte sedimentation rate (ESR), and perhaps procalcitonin, as an adjunctive measure for establishing the diagnosis. (Weak; low).

#### Decision: Adopted

Rationale: The panel decided to adopt the recommendation unchanged following screening as judgements were consistent with the IWGDF and the recommendation was considered acceptable and applicable in the Australian setting (Table 1).

#### Summary justification

The panel agreed with the IWGDF that there was a low quality of evidence and a weak (strength of) recommendation for the recommendation given the minimal evidence supporting this recommendation. The recommendation was considered compatible and applicable to the Australian context, with the appropriate expertise and resources available to undertake these tests in many locations, although it was noted that procalcitonin had the most barriers to use.

#### Implementation considerations

Consistent with the evidence that CRP correlates most consistently and strongly with infection severity and changes rapidly, enabling dynamic interpretation, CRP was considered likely to be the most useful biomarker in this setting for many Australian settings. Procalcitonin is not widely available in the Australian healthcare setting and many clinicians likely have a decreased understanding of when it is best utilised, and its interpretation compared with CRP and ESR. In addition, it may incur additional costs in some settings and there may be a delayed time to results. The panel noted that despite the correlation between ESR and infection, it is generally used less commonly than CRP by Australian Infectious Diseases physicians.

#### Subgroup considerations

##### Geographically remote people

The panel noted that measurement of inflammatory biomarkers may not be available in all locations in a timely and feasible manner preventing implementation of this recommendation. As noted in the implementation section, access or delayed reporting is likely to be most widespread for procalcitonin.

##### Aboriginal and Torres Strait islander peoples

Similar to people in geographically remote locations it was noted that some Aboriginal and Torres Strait Islander Peoples may be located in remote areas restricting access to and delaying reporting of biomarkers. The panel noted that some Aboriginal and Torres Strait Islander Peoples may object to blood being taken, highlighting the need for clinicians to consider the necessity of testing for biomarkers (or other blood testing) and the need to explain the importance of this testing when required. There should also be consideration of language barriers as described in Recommendation 2.

#### Future research considerations

The IWGDF identified a need for additional research to correlate biomarkers with severity of infection, with careful identification of individuals who had been pre-treated with antibiotics. This was supported by the panel.

## Question two part b

In a person with diabetes and a suspected foot infection, do the IWGDF/IDSA criteria for diagnosing soft tissue infection correlate with results of skin temperature measurement or quantitative microbiology?

### Recommendation 4

As neither electronically measuring foot temperature nor using quantitative microbial analysis has been demonstrated to be useful as a method for diagnosing diabetes-related foot infection, we suggest not using them. (Weak; low).

#### Decision: Adopted

Rationale: The panel decided to adopt the recommendation unchanged following screening as judgements were consistent with the IWGDF and the recommendation was considered acceptable and applicable in the Australian setting (Table 1).

#### Summary justification

The panel agreed with the IWGDF that there was a low quality of evidence for the use of foot temperature and quantitative microbial analysis to diagnose diabetes-related foot infection. They agreed that a weak (strength of) recommendation against their use was appropriate and consistent with minimal to no benefits, their low availability and in the case of quantitative microbial analysis, the associated potential delays and expense. The lack of resources and expertise recognised in the IWGDF guidelines also exist in the Australian setting. Thus, the recommendation against their use was considered applicable to the values of Australian patients.

#### Implementation considerations

As identified by the IWGDF there is low availability of these techniques in the global context and quantitative microbial analysis is time-consuming and expensive. These considerations are also pertinent in Australia.

#### Subgroup considerations

##### Geographically remote people

The panel considers the barriers identified to implementation in the Implementation considerations section even more relevant to geographically remote peoples.

##### Aboriginal and Torres Strait islander peoples

The panel considers the barriers identified to implementation in the Implementation considerations section even more relevant to many Aboriginal and Torres Strait Islander Peoples.

#### Future research considerations

As identified by the IWGDF there is a need for further evaluation of infrared imaging when coupled to photographic assessment through telemedicine. Similarly, the role of quantitative and semi-quantitative microbial analysis in clinical management of diabetes-related foot infections needs further exploration. This will require consistent methodology such as consensus definitions for the presence of infection as well as procedures for collection and processing of samples.

## Question three

In a person with diabetes and suspected bone infection of the foot, which diagnostic tests best correlate with the presence of osteomyelitis, as diagnosed based on culture and/or histopathology of a bone specimen?

### Recommendation 5

In a person with diabetes and suspected osteomyelitis of the foot, we recommend using a combination of the probe-to-bone test, the erythrocyte sedimentation rate (or C-reactive protein and/or procalcitonin), and plain X-rays as the initial studies to diagnose osteomyelitis. (Strong; moderate).

#### Decision: Adopted

Rationale: The panel decided to adopt the recommendation unchanged following screening as judgements were consistent with the IWGDF and the recommendation was considered acceptable and applicable in the Australian setting (Table 1).

#### Summary justification

The panel agreed with the IWGDF that there was a moderate quality of evidence and a strong (strength of) recommendation for the recommendation. There is considerable evidence to support the use of the probe to bone test, ESR and plain X-rays to diagnose osteomyelitis in particular. The recommendation was considered compatible and applicable to the Australian context, with the appropriate expertise and resources available to undertake these tests in many locations, although it was noted that procalcitonin had the most barriers to use.

#### Implementation considerations

As described in the implementation section in Recommendation 3 procalcitonin is not widely available in the Australian healthcare setting, has less clinician expertise and in some settings may be associated with increased costs and delayed time to results. There is the potential to increase teaching of the probe to bone test, which is inexpensive and easy to learn and perform, to a broader clinical group.

#### Subgroup considerations

##### Geographically remote people

The panel noted that measurement of inflammatory biomarkers and plain X-rays may not be available in all locations in a timely and feasible manner. As noted in the implementation section, access or delayed reporting is likely to be most widespread for procalcitonin.

##### Aboriginal and Torres Strait islander peoples

Similar to people in geographically remote locations it was noted that some Aboriginal and Torres Strait Islander Peoples may be located in remote areas restricting access to and delaying reporting of diagnostic results. The panel also noted that some Aboriginal and Torres Strait Islander Peoples may object to bone being taken, highlighting the need for clinicians to consider the necessity of bone biopsy (or other specimens) and the need to explain the importance of this testing when required. There should also be consideration of language barriers as described in Recommendation 2.

#### Future research considerations

The panel identified that larger studies are needed to identify the sensitivity and specificity of the described tests to identify osteomyelitis. Additional studies assessing teaching and implementation of the probe to bone test to improve interobserver reliability were also recommended.

### Recommendation 6a

In a person with diabetes and suspected osteomyelitis of the foot, if a plain X-ray and clinical and laboratory findings are most compatible with osteomyelitis, we recommend no further imaging of the foot to establish the diagnosis. (Strong; low).

#### Decision: Adopted

Rationale: The panel decided to adopt the recommendation unchanged following screening as judgements were consistent with the IWGDF and the recommendation was considered acceptable and applicable in the Australian setting (Table 1).

#### Summary justification

The panel agreed with the IWGDF that there was a low quality of evidence but a strong (strength of) recommendation for the recommendation given the lack of need for more expensive and less available imaging if a diagnosis is evident. The recommendation was considered compatible with most patients’ values, applicable to the Australian context and feasible in most Australian locations, although it was noted that in some patients additional imaging may be needed to determine the extent or complications, and thus the management of the osteomyelitis.

#### Subgroup considerations

##### Geographically remote people

The panel noted that plain X-rays and laboratory assessment may not be available in remote locations in a timely and feasible manner.

##### Aboriginal and Torres Strait islander peoples

Similar to people in geographically remote locations it was noted that some Aboriginal and Torres Strait Islander Peoples may be located in remote locations restricting access to plain X-rays and laboratory assessment.

#### Future research considerations

The panel noted that additional research could be undertaken to determine the sensitivity and specificity of a panel of low cost clinical, laboratory and radiological methods to diagnose osteomyelitis.

### Recommendation 6b

If the diagnosis of osteomyelitis remains in doubt, consider ordering an advanced imaging study, such as magnetic resonance imaging (MRI) scan, 18F-FDG-positron emission tomography (PET)/computed tomography (CT) or leukocyte scintigraphy (with or without CT). (Strong; moderate).

#### Decision: Adopted

Rationale: The panel decided to adopt the recommendation unchanged following screening as judgements were consistent with the IWGDF and the recommendation was considered acceptable and applicable in the Australian setting (Table 1).

#### Summary justification

The panel agreed with the IWGDF that there was a moderate quality of evidence and a strong (strength of) recommendation for the recommendation. There is considerable evidence to support the use of these investigations to diagnose osteomyelitis which all demonstrate good sensitivity and specificity. The recommendation was considered compatible and applicable to the Australian context, with the appropriate expertise and resources available to undertake these tests in most tertiary healthcare settings in Australia.

#### Implementation considerations

The panel noted that the imaging techniques are generally available in tertiary healthcare settings in Australia, however, they are regularly not available in rural and remote settings. Furthermore, PET-CT is not reimbursed through the Pharmaceutical Benefits Scheme for this indication, restricting accessibility to those unable or unwilling to pay the associated costs.

#### Subgroup considerations

##### Geographically remote people

The panel noted that the imaging techniques are regularly not available in rural and remote settings, restricting access unless patients travel to a tertiary centre.

##### Aboriginal and Torres Strait islander peoples

Similar to people in geographically remote locations it was noted that some Aboriginal and Torres Strait Islander Peoples may be located in remote areas restricting access to the imaging techniques.

### Recommendation 7

In a person with diabetes and suspected osteomyelitis of the foot, in whom making a definitive diagnosis or determining the causative pathogen is necessary for selecting treatment, collect a sample of bone (percutaneously or surgically) to culture clinically relevant bone microorganisms and for histopathology (if possible). (Strong; low).

#### Decision: Adopted

Rationale: The panel decided to adopt the recommendation unchanged following screening as judgements were consistent with the IWGDF and the recommendation was considered acceptable and applicable in the Australian setting (Table 1).

#### Summary justification

The panel agreed with the IWGDF that there was a low quality of evidence but a strong (strength of) recommendation for the recommendation given that biopsy is considered the gold-standard for diagnosis of osteomyelitis and there is substantial benefit of using directed antibiotic therapy in individuals with recalcitrant infection or in those that are at higher risk of antibiotic resistance. The recommendation was considered compatible with most patients’ values given the increased chance of cure of infection, however, it was noted that there is the theoretical risk of introducing infection and a potential risk of fracture. The recommendation was considered applicable to the Australian context and feasible in most Australian locations, although expertise for percutaneous biopsy was noted to be low in many Australian locations.

#### Implementation considerations

The panel noted that the availability of percutaneous bone biopsy is variable across the Australian healthcare system. In many locations, experience and skill with this technique are not available, which potentially reduces the ability to implement this recommendation.

#### Subgroup considerations

##### Geographically remote people

The patchy availability of percutaneous biopsy identified in the Implementation section is considered likely to be greater in rural and remote locations. Furthermore, in remote locations there is reduced access to specialist surgical services to enable surgical biopsy and there may be delays in the time to results of microbiological and histological specimens.

##### Aboriginal and Torres Strait islander peoples

It was noted that some Aboriginal and Torres Strait Islander Peoples may be located in remote locations and encounter the same issues identified for people in geographically remote locations. The panel also noted that some Aboriginal and Torres Strait Islander Peoples may object to bone being taken as described in Recommendation 5. There should also be consideration of language barriers as described in Recommendation 2.

#### Future research considerations

The panel identified that research on the availability and usage of percutaneous bone biopsy in patients with diabetes-related foot osteomyelitis across Australia would enable an assessment of current expertise and usage and inform future education and implementation of this procedure.

## Question four

In a person with diabetes and a foot infection, do specimens of wound tissue (obtained by curettage or biopsy) provide more clinically useful information on growth of pathogens or avoidance of contaminants than wound swabs?

### Recommendation 8a

Collect an appropriate specimen for culture for almost all clinically infected wounds to determine the causative pathogens. (Strong; low).

#### Decision: Adopted

Rationale: The panel decided to adopt the recommendation unchanged following screening as judgements were consistent with the IWGDF and the recommendation was considered acceptable and applicable in the Australian setting (Table 1).

#### Summary justification

The panel agreed with the IWGDF that there was a low quality of evidence but a strong (strength of) recommendation for the recommendation given the benefit of identifying the microbiological cause of infection in directing antibiotic therapy. Culture was noted to not only be important for pathogen identification but also to determine the susceptibility profile of these pathogens. The recommendation was considered compatible with most patients’ values, applicable to the Australian context and feasible in most Australian locations.

#### Subgroup considerations

##### Geographically remote people

The panel noted that there will be a potential delay in time to results in microbiological testing in some remote locations.

##### Aboriginal and Torres Strait islander peoples

Some Aboriginal and Torres Strait Islander Peoples located in remote locations may encounter delayed time to results similar to people in geographically remote locations. The panel also noted that some Aboriginal and Torres Strait Islander Peoples may object to tissue being taken as described in Recommendation 5. There should also be consideration of language barriers as described in Recommendation 2.

#### Monitoring considerations

It is suggested that services consider recording whether microbiological specimens are taken for each patient with a diabetes-related foot infection, to enable evaluation of the proportion of patients receiving microbiological diagnoses.

#### Future research considerations

The panel noted that studies to assess infection outcomes in patients from remote locations where access to microbiology diagnosis is difficult could be undertaken to compare treatment with directed antibiotic therapy versus treatment with empiric antibiotic therapy.

### Recommendation 8b

For a soft tissue diabetes-related foot infection, obtain a sample for culture by aseptically collecting a tissue specimen (by curettage or biopsy) from the ulcer. (Strong; moderate).

#### Decision: Adopted

Rationale: The panel decided to adopt the recommendation unchanged following screening as judgements were consistent with the IWGDF and the recommendation was considered acceptable and applicable in the Australian setting (Table 1).

#### Summary justification

The panel agreed with the IWGDF that there was a moderate quality of evidence and a strong (strength of) recommendation for the recommendation. In general tissue biopsy is the most appropriate specimen for soft tissue diabetes-related foot infections, given that biopsy is considered the gold-standard for diagnosis of infection and there is substantial benefit of using directed antibiotic therapy in individuals with recalcitrant infection or in those that are at higher risk of antibiotic resistance. The recommendation was considered compatible with most patients’ values, applicable to the Australian context and feasible in most Australian locations.

#### Implementation considerations

The panel noted that although expertise for soft tissue biopsy is available in most Australian healthcare settings, this may be reduced in some rural and remote settings. Furthermore, in some locations, tissue specimens may potentially be processed more slowly than tissue swabs.

#### Subgroup considerations

##### Geographically remote people

The panel noted that there may be reduced expertise in soft tissue biopsy and a potential delay in time to results in microbiological testing in some rural and remote locations.

##### Aboriginal and Torres Strait islander peoples

Similar issues to those identified for geographically remote populations may exist for Aboriginal and Torres Strait Islander Peoples located in remote locations. The panel also noted that some Aboriginal and Torres Strait Islander Peoples may object to tissue being taken as described in Recommendation 5. There should also be consideration of language barriers as described in Recommendation 2.

##### Monitoring considerations

It is suggested that services consider recording whether tissue specimens are taken for each patient with a diabetes-related foot infection, and the type of specimen collected, to enable evaluation of the proportion of patients receiving microbiological diagnoses based on tissue samples compared with superficial swabs.

#### Future research considerations

The panel noted that additional studies are needed to identify the likelihood of deep infection caused by a specific organism identified on a superficial swab. Furthermore, the panel suggests that the relative benefit of tissue samples versus swabs be assessed in clinical trials looking at patient outcomes.

## Question five

In a person with diabetes and a foot infection, do the results of molecular (genotypic) microbiological tests better distinguish likely clinically relevant pathogens requiring antibiotic therapy than standard (phenotypic) cultures?

### Recommendation 9

Do not use molecular microbiology techniques (instead of conventional culture) for the first-line identification of pathogens from samples in a patient with a diabetes-related foot infection. (Strong; low).

#### Decision: Adopted

Rationale: The panel decided to adopt the recommendation unchanged following screening as judgements were consistent with the IWGDF and the recommendation was considered acceptable and applicable in the Australian setting (Table 1).

#### Summary justification

The panel agreed with the IWGDF that there was a low quality of evidence and a strong (strength of) recommendation against the recommendation given the lack of evidence for benefit of molecular microbiology techniques. The recommendation was considered compatible with patients’ values, applicable to the Australian context and feasible in most Australian locations given molecular microbiology techniques are rarely available.

#### Implementation considerations

There is low availability of molecular microbiology in the Australian context and the techniques are still relatively new and expensive.

#### Subgroup considerations

##### Geographically remote people

The panel considers the barriers identified to implementation in the Implementation considerations section even more relevant to geographically remote peoples.

##### Aboriginal and Torres Strait islander peoples

The panel considers the barriers identified to implementation in the Implementation considerations section even more relevant to many Aboriginal and Torres Strait Islander Peoples.

#### Future research considerations

The panel noted that there is a need for further development and investigation of molecular microbiology techniques including studies that compare results with standard cultures according to IWGDF grade of infection and assess patient-related outcomes.

## Question six

In a person with diabetes and a foot infection, is any particular antibiotic regimen (specific agent [s], route, duration) better than any other for treating soft tissue or bone infection?

### Recommendation 10

Treat a person with a diabetes-related foot infection with an antibiotic agent that has been shown to be effective in a published randomised controlled trial and is appropriate for the individual patient. Some agents to consider include penicillins, cephalosporins, carbapenems, metronidazole (in combination with other antibiotic [s]), clindamycin, linezolid, daptomycin, fluoroquinolones, or vancomycin, but not tigecycline. (Strong; high).

#### Decision: Adopted

Rationale: The panel decided to adopt the recommendation unchanged following screening as judgements were consistent with the IWGDF and the recommendation was considered acceptable and applicable in the Australian setting (Table 1).

#### Summary justification

The panel agreed with the IWGDF that there was a high quality of evidence and a strong (strength of) recommendation for the recommendation. Although evidence is based on good quality randomised controlled trials (RCTs), it was noted that the majority of these demonstrated that agents were non-inferior to each other, raising the possibility that other agents that have not been tested in RCTs may be similarly effective (i.e. agents such as cotrimoxazole and doxycycline which are commonly used for diabetes-related foot infections in Australia). The recommendation was considered compatible and applicable to the Australian context, where there is appropriate expertise and resources available to use these antibiotics in most primary, secondary and tertiary healthcare settings. The recommendation was consistent with existing Australian antibiotic guidelines [33].

#### Implementation considerations

The panel noted that multiple additional factors are important in determining the appropriate antibiotic to use for a patient, similar to those described in Recommendation 11, including severity of infection, route of administration, adverse drug reactions, current and prior microbiological results, local antibiotic resistance patterns, appropriate antimicrobial stewardship, antibiotic restrictions, cost and access. Specifically, some antibiotics will only be available in tertiary settings, and even then, more restricted antibiotics such as daptomycin may not be widely accessible.

#### Subgroup considerations

##### Geographically remote people

The panel noted that the use of intravenous antibiotics may be difficult in some rural and remote locations, requiring patient transfer to a tertiary centre.

##### Aboriginal and Torres Strait islander peoples

Similar to people in geographically remote locations it was noted that some Aboriginal and Torres Strait Islander Peoples may be located in remote areas restricting access to intravenous antibiotics.

#### Monitoring considerations

The panel recommends that individual services should collaborate with their local antimicrobial stewardship team to evaluate their local antibiotic usage and compare it to similar services and centres where possible.

#### Future research considerations

The panel noted there is a need for studies comparing regularly used empiric antibiotic regimens (rather than new antibiotics) in order to identify the best empiric regimen for different severity infections.

### Recommendation 11

Select an antibiotic agent for treating a diabetes-related foot infection based on: the likely or proven causative pathogen(s) and their antibiotic susceptibilities; the clinical severity of the infection; published evidence of efficacy of the agent for diabetes-related foot infections; risk of adverse events, including collateral damage to the commensal flora; likelihood of drug interactions; agent availability; and, financial costs. (Strong; moderate).

#### Decision: Adopted

Rationale: The panel decided to adopt the recommendation unchanged following screening as judgements were consistent with the IWGDF and the recommendation was considered acceptable and applicable in the Australian setting (Table 1).

#### Summary justification

The panel agreed with the IWGDF that there was a moderate quality of evidence and a strong (strength of) recommendation for the recommendation. The recommendation was considered compatible with most patients’ values, applicable to the Australian context and feasible in most Australian locations. It was noted that the list of considerations in the recommendation is not exhaustive and there are many additional patient-related considerations including patient acceptance of antibiotic frequency or administration type, patient adherence to a regimen, and patient preference to be treated in the outpatient setting or on country where possible. In addition, there should be a preference for narrower spectrum antibiotics where possible from an antimicrobial stewardship perspective. Conversely, at times it may be appropriate to use broader spectrum antibiotics if there is a history of recent infection or colonisation with multidrug resistant organisms or if local antimicrobial susceptibility profiles demonstrate an increased risk of such organisms.

#### Implementation considerations

As described in the Summary justification, the panel identified a number of additional implementation considerations, many of which are patient-related. These are described in additional detail in the Subgroup considerations below.

#### Subgroup considerations

##### Geographically remote people

The panel noted that people in geographically remote locations may have a greater preference to be treated in the outpatient setting to avoid travel away from home. This led to variation in the importance that they place on the use of some antibiotics over others. For example, they may prefer to trial oral antibiotics rather than intravenous antibiotics.

##### Aboriginal and Torres Strait islander peoples

The panel noted that Aboriginal and Torres Strait Islander Peoples may have a greater preference to be treated in the outpatient setting with oral antibiotics or prefer to use intravenous antibiotics through outpatient parenteral services if available to enable them to stay on country or avoid inpatient hospital admissions. There should also be consideration of language barriers as described in Recommendation 2.

##### Other subgroup considerations

The panel noted that an increased preference to be treated in the outpatient setting with oral antibiotics or use intravenous antibiotics through outpatient parenteral services may exist for many other patient groups, including carers and those with dependants. In addition, broader spectrum antibiotics may be commenced if patients have a history of recent infection or colonisation with multidrug resistant organisms (such as methicillin-resistant *Staphylococcus aureus* (MRSA)) or if local antimicrobial susceptibility profiles demonstrate an increased risk of multidrug resistant organisms. Risk factors for MRSA infection in the Australian context have been described [34].

#### Future research considerations

The panel noted that qualitative studies to explore and rank the factors most important for patients would assist clinicians in understanding patients’ preferences and providing the most balanced options when discussing treatment with patients. In addition, preferences could be assessed for specific patient subgroups such as those in geographically remote locations or Aboriginal and Torres Strait Islander Peoples.

### Recommendation 12

Administer antibiotic therapy initially by the parenteral route to any patient with a severe (grade 4) skin and soft tissue diabetes-related foot infection. Switch to oral therapy if the patient is clinically improving and has no contraindications to oral therapy and if there is an appropriate oral agent available. (Strong; very low).

#### Decision: Adopted

Rationale: The panel decided to adapt this recommendation after full assessment based on having differences in judgements to IWGDF for quality of evidence (Table 2) and due to a lack of clarity around the population it referred to. The changes made to the original IWGDF recommendation included downgrading the quality of evidence from low to “very low” and including the phrase “skin and soft tissue” to define the relevant population of patients with diabetes-related foot infection (Table 3).

#### Summary justification

Although the panel downgraded the quality of evidence to very low, they agreed with the IWGDF that diabetes-related foot infections were an important health problem in Australia, and that the balance of effects favoured the use of initial intravenous antibiotics for severe (grade 4) skin and soft tissue diabetes-related foot infections. It was noted that a switch to oral therapy when the patient was clinically improving was appropriate for severe (grade 4) skin and soft tissue infections. Furthermore, it was noted that in the IWGDF guidelines this recommendation was intended to relate to people with skin and soft tissue infections, sitting under a sub-heading stating this, however, this was not clear when the recommendation was read outside the context of the overall IWGDF guideline document. The panel were unsure whether the critical outcome of clinical cure of infection would be consistently valued above all other outcomes by all patients (for example some patients may prefer avoidance of amputation and long term antibiotic suppression). They noted that the recommendation was likely acceptable and feasible in the Australian setting. Detailed justifications from the panel’s full assessment are provided in Supplementary Table S1.

#### Subgroup considerations

##### Geographically remote people

Individuals in geographically remote populations may require initial intramuscular administration of antibiotics or once off intravenous antibiotics before transfer to a larger facility. As such, treatment may be unable to be undertaken in a remote location. For the majority of individuals, the potential for clinical cure facilitated through such a transfer would likely outweigh the potential suboptimal care in a less equipped environment and the need for transfer.

##### Aboriginal and Torres Strait islander peoples

Aboriginal and Torres Strait Islander Peoples living in remote locations are likely to require similar considerations to people living in geographically remote locations.

#### Future research considerations

One of the key research priorities identified by IWGDF was whether oral antibiotic therapy alone is as effective as parenteral treatment for diabetes-related foot infections, including diabetes-related foot osteomyelitis. The panel noted a need for studies to evaluate whether all patients with severe (grade 4) infections require initial parenteral antibiotic therapy. Furthermore, they identified a need for studies to explore the duration of initial parenteral antibiotics that is needed prior to oral switch for patients with severe (grade 4) diabetes-related foot infections and the factors that influence this decision.

### Recommendation 13

Treat patients with a mild (grade 2) diabetes-related foot infection, and most with a moderate (grade 3) diabetes-related foot infection, with oral antibiotic therapy, either at presentation or when clearly improving with initial intravenous therapy. (Weak; low).

#### Decision: Adopted

Rationale: The panel decided to adopt the recommendation unchanged following screening as judgements were consistent with the IWGDF and the recommendation was considered acceptable and applicable in the Australian setting (Table 1).

#### Summary justification

The panel agreed with the IWGDF that there was a low quality of evidence and a weak (strength of) recommendation for the recommendation. The recommendation was considered compatible with patients’ values, applicable to the Australian context and feasible in most Australian locations.

#### Future research considerations

IWGDF highlighted a need to further understand whether complete oral therapy is as effective as parenteral treatment for diabetes-related foot infections. The panel agreed that studies are needed to compare the use of complete oral therapy with initial intravenous therapy in infections of moderate (grade 3) severity and that they should assess patient outcomes.

### Recommendation 14

We suggest not using any currently available topical antimicrobial agent for treating a mild (grade 2) diabetes-related foot infection. (Weak; moderate).

#### Decision: Adopted

Rationale: The panel decided to adopt the recommendation unchanged following screening as judgements were consistent with the IWGDF and the recommendation was considered acceptable and applicable in the Australian setting (Table 1).

#### Summary justification

The panel agreed with the IWGDF that when specifically considering the use of topical antibiotic agents for mild bacterial infection there was a moderate quality of evidence and a weak (strength of) recommendation against using currently available topical antimicrobial agents given a lack of evidence demonstrating efficacy of these agents, and their potential to increase the risk of antimicrobial resistance. The panel also noted that the use of anti-septic agents is considered separately in recommendation 27b. The recommendation was considered compatible with most patients’ values, and applicable and feasible in the Australian setting.

#### Future research considerations

The panel noted that topical antimicrobial agents remain an important area of future research which have the potential to alter the treatment pathways of diabetes-related foot infections if efficacious and safe agents are identified.

### Recommendation 15a

Administer antibiotic therapy to a patient with a skin or soft tissue diabetes-related foot infection for a duration of 1 to 2 weeks. (Strong; high).

#### Decision: Adopted

Rationale: The panel decided to adopt the recommendation unchanged following screening as judgements were consistent with the IWGDF and the recommendation was considered acceptable and applicable in the Australian setting (Table 1).

#### Summary justification

The panel agreed with the IWGDF that there was a high quality of evidence and a strong (strength of) recommendation for the recommendation. The recommendation was consistent with existing Australian antibiotic guidelines [33], and considered compatible with patients’ values, applicable to the Australian context and feasible in primary, secondary and tertiary healthcare settings in Australia.

#### Monitoring considerations

The panel recommends that services record the duration of antibiotic treatment provided to patients to enable an audit of treatment duration by infection severity compared with the guidelines.

#### Future research considerations

The IWGDF identified a need for further studies to determine the optimal duration of treatment for skin and soft tissue infections. The panel noted that such studies should be categorised by infection severity and infecting microorganisms and should consider additional confounders such as severe peripheral artery disease.

### Recommendation 15b

Consider continuing treatment, perhaps for up to 3 to 4 weeks, if the infection is improving but is extensive and is resolving slower than expected or if the patient has severe peripheral artery disease. (Weak; low).

#### Decision: Adopted

Rationale: The panel decided to adopt the recommendation unchanged following screening as judgements were consistent with the IWGDF and the recommendation was considered acceptable and applicable in the Australian setting (Table 1).

#### Summary justification

The panel agreed with the IWGDF that there was a low quality of evidence and a weak (strength of) recommendation for the recommendation. The recommendation was noted to be pragmatic and generally consistent with existing Australian antibiotic guidelines [33]. It was considered compatible with patients’ values, applicable to the Australian context and feasible in primary, secondary and tertiary healthcare settings in Australia.

#### Monitoring considerations

See Recommendation 15a.

#### Future research considerations

See Recommendation 15a.

### Recommendation 15c

If evidence of infection has not resolved after 4 weeks of apparently appropriate therapy, re-evaluate the patient, and reconsider the need for further diagnostic studies or alternative treatments. (Strong; low).

#### Decision: Adopted

Rationale: The panel decided to adopt the recommendation unchanged following screening as judgements were consistent with the IWGDF and the recommendation was considered acceptable and applicable in the Australian setting (Table 1).

#### Summary justification

The panel agreed with the IWGDF that there was a low quality of evidence but a strong (strength of) recommendation for the recommendation. The recommendation was noted to be pragmatic and generally consistent with existing Australian practice. It was considered compatible with patients’ values, applicable to the Australian context and feasible in primary, secondary and tertiary healthcare settings in Australia.

#### Monitoring considerations

See Recommendation 15a.

#### Future research considerations

See Recommendation 15a.

### Recommendation 16

For patients who have not recently received antibiotic therapy and have an acute infection, consider targeting empiric antibiotic therapy at just aerobic Gram positive pathogens (beta-haemolytic streptococci and *Staphylococcus aureus*) in cases of a mild (grade 2) diabetes-related foot infection. (Weak; low).

#### Decision: Adopted

Rationale: The panel decided to adapt this recommendation after full assessment based on having differences in judgements to IWGDF for balance of effects and the population impacted (Table 2). The changes made to the original IWGDF recommendation included downgrading the balance of effects from strong to “weak”, extending the recommendation to all locations in Australia by excluding the need for patients to reside in a temperate climate area and narrowing the population by adding the phrase “and have an acute infection” (Table 3).

#### Summary justification

The panel agreed with the IWGDF that diabetes-related foot infections were an important health problem in Australia, that the use of empiric narrower spectrum antibiotics had more desirable benefits than undesirable benefits, and that the quality of the evidence supporting this was low. However, the panel felt the balance of effects was weak, consistent with a conditional recommendation for narrower spectrum antibiotics in the described circumstances and consistent with the use of the word consider in the recommendation. The panel also noted that although the recommendation was likely acceptable and feasible in the Australian setting, Australian practice and guidelines [33] do not distinguish use of narrow spectrum antibiotics by climate and there is no local evidence to support such a distinction. However, both local guidelines [33] and studies [35] support use of narrower spectrum agents in acute infections. The panel noted that the definition of acute infection in the published literature has varied from less than 2 to 6 weeks and suggest that, in concordance with local guidelines [33], duration of infective symptoms of less than 4 weeks could be considered acute while noting broader therapy may be required for those with a duration of ulceration of greater than 6 weeks [35] and in those with recent antibiotic exposure [36]. Detailed justifications are described in Supplementary Table S2.

#### Subgroup considerations

The panel noted that there is no evidence from the Australian context to suggest individuals living in tropical regions with acute infections cannot be treated with narrow spectrum antibiotics and current practice in Australia is to treat such individuals with narrow spectrum antibiotics. They also noted that in patients known to be colonised with MRSA or in areas with a high prevalence, prescribers should consider empiric coverage of MRSA. Many tropical regions of Australia are also remote and increased rates of MRSA may exist in some of these remote populations. Similarly, many Aboriginal and Torres Strait Islanders live in tropical regions of Australia and there is an increased rate of MRSA in some Aboriginal and Torres Strait Islander populations [5].

#### Future research considerations

The panel identified a need for further studies to investigate whether a difference in patient outcomes exists between patients treated with narrow compared with broad spectrum antibiotics in patients with acute infections. They also highlighted the need for further studies comparing pathogens in acute infections between temperate and tropical regions of Australia.

### Recommendation 17

For patients who have been treated with antibiotic therapy within a few weeks, have a chronic infection, have a severely ischaemic affected limb, or a moderate (grade 3) or severe (grade 4) infection, we suggest selecting an empiric antibiotic regimen that covers Gram positive pathogens, commonly isolated Gram negative pathogens, and possibly obligate anaerobes in cases of moderate (grade 3) to severe (grade 4) diabetes-related foot infections. Then, reconsider the antibiotic regimen based on both the clinical response and culture and sensitivity results. (Weak; low).

#### Decision: Adopted

Rationale: The panel decided to adapt this recommendation after full assessment based on having differences in judgements to IWGDF for the population impacted in the Australian setting (Table 2). This was achieved by extending the recommendation to all locations in Australia by excluding the need for patients to reside in a tropical/subtropical climate and including patients with chronic infections by adding the phrase “who have a chronic infection” (Table 3).

#### Summary justification

The panel agreed with the IWGDF that diabetes-related foot infections were an important health problem in Australia, that the use of empiric narrower spectrum antibiotics had more desirable benefits than undesirable benefits, that the quality of the evidence supporting this was low and the balance of effects was weak. The panel also noted that although the recommendation was likely acceptable and feasible in the Australian setting, similar to Recommendation 16, Australian practice and guidelines [33] do not distinguish use of antibiotic spectrum by climate and there is no local evidence to support such a distinction. However, both local guidelines [33] and studies [35] support use of broader spectrum agents in chronic infections and in the presence of chronic ulceration. As described in Recommendation 16, the panel noted that the definition of acute infection and thus chronic infection varies in the published literature and suggest that, in concordance with local guidelines [33], duration of infective symptoms of four or more weeks could be considered chronic while noting broader therapy may also be required for those with a duration of ulceration of greater than 6 weeks [35]. Detailed justifications are described in Supplementary Table S3.

#### Subgroup considerations

See Recommendation 16.

#### Future research considerations

See Recommendation 16.

### Recommendation 18

Empiric treatment aimed at *Pseudomonas aeruginosa* is not usually necessary but consider it if *P. aeruginosa* has been isolated from cultures of the affected site within the previous few weeks, or in tropical/subtropical climates (at least for moderate [grade 3] or severe [grade 4] infection). (Weak; low).

#### Decision: Adopted

Rationale: The panel decided to adapt this recommendation after full assessment based on minor differences in judgements to IWGDF for the population impacted in the Australian setting (Table 2). This was achieved by extending the recommendation to all locations in Australia by excluding the phrase “in temperate climates” (Table 3).

#### Summary justification

The panel agreed with the IWGDF that diabetes-related foot infections were an important health problem in Australia, that the use of empiric antibiotic treatment to cover *P. aeruginosa* in certain circumstances had more desirable benefits than undesirable benefits, that the quality of the evidence supporting this was low and the balance of effects was weak. The panel also noted that although the recommendation was likely acceptable and feasible in the Australian setting, *P. aeruginosa* can be a pathogen in temperate as well as tropical regions [35]. Detailed justifications are described in Supplementary Table S4.

#### Implementation considerations

The panel noted that in Australia, many clinicians obtain cultures via superficial swabs. Thus, increased weight should be given to treatment covering *P. aeruginosa* if it has been previously isolated from tissue samples from the affected site compared with superficial swabs.

#### Future research considerations

The panel identified a number of potential areas for future research that related to this recommendation including:

1. Studies to investigate differences in the prevalence of *P. aeruginosa* in diabetes-related foot infections in temperate and tropical regions of Australia and how this differs by severity of infection.
2. Studies to investigate differences in outcomes of diabetes-related foot infections treated with empiric *P. aeruginosa* coverage versus those that are not.
3. Studies to investigate differences in outcomes of diabetes-related foot infections that culture *P. aeruginosa* and are treated with antibiotics that target this bacteria versus those that do not.

### Recommendation 19

Do not treat clinically uninfected foot ulcers with systemic or local antibiotic therapy with the goal of reducing the risk of infection or promoting ulcer healing. (Strong; low).

#### Decision: Adopted

Rationale: The panel decided to adopt the recommendation unchanged following screening as judgements were consistent with the IWGDF and the recommendation was considered acceptable and applicable in the Australian setting (Table 1).

#### Summary justification

The panel agreed with the IWGDF that there was a low quality of evidence but a strong (strength of) recommendation for the recommendation. The recommendation was considered to be consistent with antimicrobial stewardship principles. It was considered compatible with patients’ values, applicable to the Australian context and feasible in primary, secondary and tertiary healthcare settings in Australia.

## Question seven part a

In a person with diabetes and osteomyelitis of the foot, are there circumstances in which nonsurgical (antibiotic only) treatment is as safe and effective (in achieving remission) as surgical treatment?

### Recommendation 20

Non-surgeons should urgently consult with a surgical specialist in cases of severe (grade 4) infection or of moderate (grade 3) infection complicated by extensive gangrene, necrotising infection, signs suggesting deep (below the fascia) abscess or compartment syndrome, or severe lower limb ischaemia. (Strong; low).

#### Decision: Adopted

Rationale: The panel decided to adopt the recommendation unchanged following screening as judgements were consistent with the IWGDF and the recommendation was considered acceptable and applicable in the Australian setting (Table 1).

#### Summary justification

The panel agreed with the IWGDF that there was a low quality of evidence but a strong (strength of) recommendation for the recommendation. The recommendation was considered compatible with patients’ values, applicable to the Australian context and feasible in most secondary and tertiary healthcare settings in Australia.

#### Subgroup considerations

##### Geographically remote people

The panel noted that there is disparate access to specialist diabetes-related foot surgical services across Australia. Many remote and rural locations and some regional locations may not have nearby access to such services. Such centres need to have clear referral pathways (including criteria for referral and who to contact) and access to timely advice and transfer mechanisms.

##### Aboriginal and Torres Strait islander peoples

Aboriginal and Torres Strait Islander Peoples living in remote, rural and some regional centres may not have access to specialist surgical services as described for geographically remote people.

#### Future research considerations

The panel noted that mixed methods research to identify differences between rural and metropolitan services in time between first presentation and surgery and to identify barriers to timely surgery would likely assist in improving the referral pathways and processes for diabetes-related foot infections requiring surgery.

### Recommendation 21a

In a patient with diabetes and uncomplicated forefoot osteomyelitis, for whom there is no other indication for surgical treatment, consider treating with antibiotic therapy without surgical resection of bone. (Strong; moderate).

#### Decision: Adopted

Rationale: The panel decided to adopt this recommendation after full assessment based on having no substantial differences in judgements to IWGDF.

#### Summary justification

The panel agreed with the IWGDF that there was a moderate quality of evidence and a strong (strength of) recommendation for the recommendation. The recommendation was considered compatible with patients’ values, applicable to the Australian context and feasible in primary, secondary and tertiary healthcare settings in Australia. The panel noted that according to the exclusion criteria in the sole RCT assessing this issue [37], uncomplicated forefoot osteomyelitis should be defined as osteomyelitis without severe (grade 4) infection, and without any of the following: necrotising tissue infection, bone exposed in the base of the ulcer, kidney injury, or peripheral artery disease. Detailed justifications are described in Supplementary Table S5.

#### Implementation considerations

The panel noted that the need to be able to identify an indication for surgical intervention requires a level of specialist surgical knowledge that may not be available in all locations.

#### Subgroup considerations

##### Geographically remote people

As identified in the Implementation considerations section, the panel noted that the need to be able to identify an indication for surgical intervention requires a level of specialist surgical knowledge that may be less commonly available in remote and rural locations. There is a need for services in such locations to have networks with which they can discuss cases to receive timely advice.

##### Future research considerations

The IWGDF highlighted the need for additional research to determine the optimal duration of antibiotic therapy in patients with diabetes-related foot osteomyelitis who are treated without surgery. The panel noted that additional well-designed studies on subgroups of patients with diabetes-related foot osteomyelitis would be beneficial to further characterise the expected outcomes of non-surgical versus surgical approaches in different patient groups.

### Recommendation 21b

In a patient with probable diabetes-related foot osteomyelitis with concomitant soft tissue infection, urgently evaluate the need for surgery as well as intensive post-operative medical and surgical follow-up. (Strong; moderate).

#### Decision: Adopted

Rationale: The panel decided to adopt the recommendation unchanged following screening as judgements were consistent with the IWGDF and the recommendation was considered acceptable and applicable in the Australian setting (Table 1).

#### Summary justification

The panel agreed with the IWGDF that there was a moderate quality of evidence and a strong (strength of) recommendation for the recommendation. The recommendation was considered compatible with patients’ values, applicable to the Australian context and feasible in most secondary and tertiary healthcare settings in Australia.

#### Subgroup considerations

##### Geographically remote people

As described in Recommendation 20, there is disparate access to specialist diabetes-related foot surgical services across Australia. In addition, intensive post-operative medical and surgical follow up may be difficult in some locations and may require relocation for a period of time. The use of technology such as telehealth appointments and patient-centred co-management arrangements such as joint appointments with general practitioners and specialists should be considered where feasible.

##### Aboriginal and Torres Strait islander peoples

Aboriginal and Torres Strait Islander Peoples living in remote, rural and some regional centres may not have access to specialist surgical services as described for geographically remote people.

#### Future research considerations

The panel noted that further studies assessing outcomes such as amputation, wound healing, resolution of infection and mortality are needed for comparison of surgery versus no surgery for different subgroups of patients with diabetes-related foot infections.

### Recommendation 22

Select antibiotic agents for treating diabetes-related foot osteomyelitis from among those that have demonstrated efficacy for osteomyelitis in clinical studies. (Strong; low).

#### Decision: Adopted

Rationale: The panel decided to adopt the recommendation unchanged following screening as judgements were consistent with the IWGDF and the recommendation was considered acceptable and applicable in the Australian setting (Table 1).

#### Summary justification

The panel agreed with the IWGDF that there was a low quality of evidence but a strong (strength of) recommendation for the recommendation. The recommendation was considered compatible with patients’ values, applicable to the Australian context and feasible in primary, secondary and tertiary healthcare settings in Australia. However, it was noted by the panel that there have been few clinical trials that have compared different antibiotic regimens for diabetes-related foot osteomyelitis and it would be reasonable to use antibiotics that are used for osteomyelitis that is not associated with diabetes-related foot infections.

#### Implementation considerations

As described in the Summary justification, few antibiotic regimens have been tested in clinical trials for diabetes-related foot osteomyelitis and it would be reasonable to use antibiotics that are used for osteomyelitis that is not associated with diabetes-related foot infections. Such antibiotics include beta-lactams, beta-lactam/beta-lactamase inhibitors, (fluoro) quinolones, glycopeptides and lipoglycopeptides, oxazolidinones, sulfonamides, lincosamides, rifamycins and fusidic acid [38–41]. Trials into the benefit of adjunctive rifampicin are ongoing [42]. The panel noted that a single substudy in an RCT of tigecycline versus ertapenem +/− vancomycin found tigecycline had a statistically non-significant chance of cure but a higher adverse event rate [43]. The factors identified as important in choosing an antibiotic for skin and soft tissue infections in Recommendations 10 and 11 should also be considered when choosing an antibiotic for osteomyelitis.

#### Subgroup considerations

##### Geographically remote people

The panel noted that the use of intravenous antibiotics may be difficult in some rural and remote locations, requiring patient transfer to a tertiary centre.

##### Aboriginal and Torres Strait islander peoples

Similar to people in geographically remote locations it was noted that some Aboriginal and Torres Strait Islander Peoples may be located in remote areas restricting access to intravenous antibiotics.

#### Monitoring considerations

As identified in Recommendation 10, the panel recommends that individual services should collaborate with their local antimicrobial stewardship team to evaluate their local antibiotic usage and compare it to similar services and centres where possible.

#### Future research considerations

The panel noted there is a need for studies comparing regularly used empiric antibiotic regimens (rather than new antibiotics) when culture results are unknown in order to identify the best empiric regimen for different types and severity of osteomyelitis. This should include comparison of oral-only antibiotic regimens compared with regimens with initial intravenous antibiotics. In addition, there is a need to compare different regularly used antibiotics in patients with known pathogens to determine which are the most effective.

### Recommendation 23a

Treat diabetes-related foot osteomyelitis with antibiotic therapy for no longer than 6 weeks. If the infection does not clinically improve within the first 2 to 4 weeks, reconsider the need for collecting a bone specimen for culture, undertaking surgical resection, or selecting an alternative antibiotic regimen. (Strong; moderate).

*Decision*: Excluded.

Rationale: The panel decided to exclude this recommendation after full assessment based on having substantial differences in judgements to IWGDF for the certainty of evidence and balance of effects (Table 2) and due to inclusion of a heterogeneous population. The population addressed (person with diabetes and osteomyelitis of the foot) was considered to be too heterogeneous for the recommendation to treat with antibiotic therapy for no longer than 6 weeks to be broadly applied. The highly select subgroup for which there is some evidence to support a shorter duration of therapy is targeted in Recommendation 21(a). The second sentence of the recommendation was considered to reflect expert opinion of good practice supported by extensive experience and considered to be a general principle rather than remain as an evidence-based recommendation.

#### Summary justification

The panel disagreed with the IWGDF on a number of aspects relating to this recommendation. The population of patients with diabetes-related foot osteomyelitis was considered too heterogeneous, impacting on a number of aspects of the assessment. Given the poor representation of the breadth of clinical presentations of diabetes-related foot osteomyelitis in the literature the panel downgraded the certainty of evidence to low. Similarly, the heterogeneity of diabetes-related foot osteomyelitis meant that the balance of effects was considered to vary. The panel were unsure whether the critical outcome of clinical cure of infection would be consistently valued above others by all patients (for example some patients may prefer avoidance of amputation and long term antibiotic suppression). The acceptability of the recommendation by patients and providers in the Australian setting was considered likely to vary substantially based on the subgroup of osteomyelitis being treated. The recommendation was considered likely to be feasible in the Australian setting. Detailed justifications are described in Supplementary Table S6.

#### Future research considerations

The panel noted that exclusion of this recommendation highlights the need for further well-designed studies to better characterise and define subsets of diabetes-related foot osteomyelitis and then investigate different durations of antibiotic therapy for these subgroups.

### Recommendation 23b

Treat diabetes-related foot osteomyelitis with antibiotic therapy for just a few days if there is no soft tissue infection and all the infected bone has been surgically removed. (Weak; low).

#### Decision: Adopted

Rationale: The panel decided to adopt the recommendation unchanged following screening as judgements were consistent with the IWGDF and the recommendation was considered acceptable and applicable in the Australian setting (Table 1).

#### Summary justification

The panel agreed with the IWGDF that there was a low quality of evidence and a weak (strength of) recommendation for the recommendation. The recommendation was noted to be consistent with existing Australian infection guidelines [33] where it is recommended that antibiotics are continued for 2 to 5 days after definitive surgery for osteomyelitis. It was considered compatible with patients’ values, applicable to the Australian context and feasible in primary, secondary and tertiary healthcare settings in Australia.

#### Implementation considerations

As described in the Summary justification, the panel believe that the duration that antibiotic therapy should be continued, described as ‘just a few days’ in the recommendation should be defined as 2 to 5 days. This is consistent with Australian infection guidelines and allows clinicians to await histopathology and culture results from tissue samples taken from the presumed uninfected residual bone margin to verify adequate surgical removal of infection.

#### Monitoring considerations

The panel recommends that services record the duration of antibiotic treatment provided to patients post definitive surgery and whether tissue samples were sent from the presumed clean wound post-surgical debridement.

#### Future research considerations

The panel noted that further studies on the duration of antibiotics post definitive surgery for osteomyelitis would be beneficial including identifying factors associated with poorer outcomes that may indicate a need for more prolonged antibiotic therapy. Standardisation of terminology, sampling technique and processing methods would aid research goals.

### Recommendation 24

For people with diabetes-related foot osteomyelitis that initially require parenteral therapy, consider switching to an oral antibiotic regimen that has high bioavailability after perhaps 5 to 7 days, if the likely or proven pathogens are susceptible to an available oral agent and the patient has no clinical condition precluding oral therapy. (Weak; moderate).

#### Decision: Adopted

Rationale: The panel decided to adopt the recommendation unchanged following screening as judgements were consistent with the IWGDF and the recommendation was considered acceptable and applicable in the Australian setting (Table 1).

#### Summary justification

The panel agreed with the IWGDF that there was a moderate quality of evidence and a weak (strength of) recommendation for considering transition to oral antibiotics after 5 to 7 days. The panel noted that uncertainty remains regarding the best timing of switch to oral agents and that clinician practice may differ. This recommendation reflects a change from previous practice and Australian guidelines but is consistent with emerging evidence suggesting early switch to bioavailable oral agents is equally efficacious to longer antibiotic therapy [44]. It is likely that some of the current uncertainty around best practice will be resolved in the short to medium term as further evidence and experience of early oral switch for bone and joint infections becomes available. It was considered applicable to the Australian context and feasible in most secondary and tertiary healthcare settings in Australia. The panel also noted that an earlier switch to oral agents would be considered preferable for most patients if clinically appropriate and as such the recommendation was considered compatible with patients’ values.

#### Implementation considerations

The panel noted that earlier transition to oral agents would potentially allow patients to return home more quickly and reduce the challenges that are associated with treatment.

#### Subgroup considerations

##### Geographically remote people

As described in the Implementation considerations, earlier transition to oral agents would potentially allow patients to return home more quickly, reducing the time that patients from remote locations need to remain away from home or an inpatient. This benefit would likely be valued highly by many people in remote locations.

##### Aboriginal and Torres Strait islander peoples

As described in the Implementation considerations, earlier transition to oral agents would potentially allow Aboriginal and Torres Strait Islander Peoples to return home to country more quickly, reducing the time that patients are away from or an inpatient. This benefit would likely be valued highly by many Aboriginal and Torres Strait Islander Peoples.

#### Monitoring considerations

The panel recommends that individual services record the duration of intravenous and oral antibiotic therapy to allow comparison of patient outcomes and between services.

#### Future research considerations

The panel notes that this remains an area of active research and there is a need for further randomised trials to compare antibiotic regimens with early oral transition with longer intravenous regimens for diabetes-related foot osteomyelitis in different patient subgroups. Selection of effective oral agents requires a particular focus to understand the relative importance of bioavailability, bone penetration, biofilm activity and therapeutic drug monitoring.

## Question seven part b

In a person with diabetes and osteomyelitis of the foot who is undergoing foot surgery, is obtaining biopsy of the presumed uninfected residual bone margin useful for determining the need for additional anti-infective treatment?

### Recommendation 25a

During surgery to resect bone for diabetes-related foot osteomyelitis, consider obtaining a specimen of bone for culture (and, if possible, histopathology) at the stump of the resected bone to identify if there is residual bone infection. (Weak; moderate).

#### Decision: Adopted

Rationale: The panel decided to adopt the recommendation unchanged following screening as judgements were consistent with the IWGDF and the recommendation was considered acceptable and applicable in the Australian setting (Table 1).

#### Summary justification

The panel agreed with the IWGDF that there was a moderate quality of evidence and a weak (strength of) recommendation for the recommendation. It was considered compatible with patients’ values, applicable to the Australian context and feasible in most tertiary healthcare settings in Australia.

#### Monitoring considerations

The panel recommends that services record whether tissue samples were sent from the presumed clean wound post-surgical debridement.

#### Future research considerations

The panel noted that additional studies comparing outcomes in patients with positive and negative stump cultures would be beneficial.

### Recommendation 25b

If an aseptically collected culture specimen obtained during the surgery grows pathogen(s), or if the histology demonstrates osteomyelitis, administer appropriate antibiotic therapy for up to 6 weeks. (Strong; moderate).

#### Decision: Adopted

Rationale: The panel decided to adopt this recommendation after full assessment based on having no substantial differences in judgements to IWGDF.

#### Summary justification

The panel agreed with the IWGDF that there was a moderate quality of evidence and a strong (strength of) recommendation to continue antibiotics if proximal surgical samples suggested residual infection. The recommendation was considered compatible with patients’ values, applicable to the Australian context and feasible in secondary and tertiary healthcare settings in Australia. Detailed justifications are described in Supplementary Table S7.

#### Subgroup considerations

##### Geographically remote people

The panel noted that there may be longer turn-around times for laboratory results from bone sampling (microbiology and anatomical pathology) when sent from remote sites and that this should be considered in the treatment algorithm. Antibiotics should be continued until the results are available.

#### Monitoring considerations

See Recommendation 25a.

#### Future research considerations

The IWGDF highlighted that there is a lack of an agreed definition of osteomyelitis in the diabetes-related foot, highlighting the need for consensus definitions of [1] stages of diabetes-related foot osteomyelitis and [2] outcome assessment, in addition to standardised collection and reporting methods for bone samples. The panel noted that additional studies comparing outcomes in patients with positive and negative stump cultures would be beneficial. In addition, there is a need for studies to investigate the optimal duration of antibiotics in patients with diabetes-related foot osteomyelitis treated with bone resection surgery.

## Question eight

In a person with diabetes and a foot infection, does the addition of any specific adjunctive treatment to systemic antibiotic therapy improve resolution of clinical findings of infection or accelerate ulcer healing?

### Recommendation 26

For a diabetes-related foot infection, do not use hyperbaric oxygen therapy or topical oxygen therapy as an adjunctive treatment if the only indication is specifically for treating the infection. (Weak; low).

#### Decision: Adopted

Rationale: The panel decided to adopt the recommendation unchanged following screening as judgements were consistent with the IWGDF and the recommendation was considered acceptable and applicable in the Australian setting (Table 1).

#### Summary justification

The panel agreed with the IWGDF that there was a low quality of evidence and a weak (strength of) recommendation against the use of hyperbaric oxygen or topical oxygen therapy for treating infection. The recommendation was considered compatible with patients’ values, applicable to the Australian context and feasible in tertiary healthcare settings in Australia.

#### Implementation considerations

The panel noted that hyperbaric oxygen therapy is not widely available throughout Australia and is generally only available in selected metropolitan and regional centres.

#### Future research considerations

The panel noted that there is a need for further randomised studies with infection outcomes to assess the role of hyperbaric oxygen therapy in diabetes-related foot infections.

### Recommendation 27a

To specifically address infection in a diabetes-related foot ulcer do not use adjunctive granulocyte colony stimulating factor treatment (Weak; moderate).

#### Decision: Adopted

Rationale: The panel decided to adopt the recommendation unchanged following screening as judgements were consistent with the IWGDF and the recommendation was considered acceptable and applicable in the Australian setting (Table 1).

#### Summary justification

The panel agreed with the IWGDF that there was a moderate quality of evidence and a weak (strength of) recommendation against the use of adjunctive granulocyte colony stimulating factor treatment for diabetes-related foot infection. It was considered compatible with patients’ values, applicable to the Australian context and feasible in primary, secondary and tertiary healthcare settings in Australia.

### Recommendation 27b

To *specifically* address infection in a diabetes-related foot ulcer do not *routinely* use topical antiseptics, silver preparations, honey, bacteriophage therapy, or negative pressure wound therapy (with or without instillation). (Weak; low).

#### Decision: Adopted

Rationale: The panel decided to adopt the recommendation unchanged following screening as judgements were consistent with the IWGDF and the recommendation was considered acceptable and applicable in the Australian setting (Table 1).

#### Summary justification

The panel agreed with the IWGDF that there was a low quality of evidence and a weak (strength of) recommendation to not use these topical therapies routinely as sole therapy or instead of antibiotics for established clinical infection. However, the panel noted that these may be used as an adjunct in combination with good wound care principles and that there may be a potential benefit to using adjunctive non-harmful traditional or complementary therapies, if only to maintain a therapeutic relationship in some patients. With these caveats, the recommendation was considered compatible with patients’ values, applicable to the Australian context and feasible in most primary, secondary and tertiary healthcare settings in Australia.

#### Implementation considerations

The panel noted that while these therapies should not be used as the primary treatment for treating infection, many clinicians use these therapies as an adjunct to wound care and as they generally have minimal evidence for efficacy or harm they may be considered following discussion between the patients and clinician. Further implementation considerations are detailed in the Subgroup considerations section below.

#### Subgroup considerations

##### Aboriginal and Torres Strait islander peoples

The panel noted that some Aboriginal and Torres Strait Islanders may prefer to use a combination of traditional medicine (i.e. honey) and western medicine and that clinicians should recognise the importance of maintaining traditional practices where possible. Even without published evidence for their use, there may be additional benefits beyond those that are directly related to improving the infection, including the development of a supportive therapeutic relationship. The clinician should raise the potential for ceasing traditional medicines if there are concerns about drug interactions or adverse events.

##### Other subgroup considerations

As described for Aboriginal and Torres Strait Islander Peoples, the panel noted that some people may wish to use complementary medicine or adjunctive wound healing measures in combination with western medicine and that clinicians should recognise that there may be additional benefits beyond those that are directly related to improving the infection, including the development of a supportive therapeutic relationship. The clinician should raise the potential for ceasing complementary medicines if there are concerns about drug interactions, adverse events and/or inappropriate financial costs.

#### Monitoring considerations

The panel suggests that the use of such therapies should be recorded by each service and a regular evaluation performed to review patient outcomes and adverse events in those using such therapies.

#### Future research considerations

The panel noted that there is a need for additional high quality studies of adjunctive topical treatments in diabetes-related foot infections to determine whether there is added benefit from these therapies.

## Discussion

### Recommendations summary

Best-practice adaptation of the 2019 IWGDF Working Group’s Infection Guidelines for the Australian national context was undertaken by an expert panel, leading to the development of the first multi-disciplinary, evidence-based Australian diabetes-related foot infection guideline since 2011. A total of 27 recommendations, including 36 sub-recommendations, were screened, with 29 sub-recommendations adopted without further review and seven undergoing full assessment. Of the seven undergoing full assessment, four were adapted, two were adopted and one was excluded. All 36 original sub-recommendations were further assessed for specific considerations related to their implementation, specific subgroups (including Aboriginal and Torres Strait Islander and geographically remote populations), monitoring and future research.

Adaptation of an existing guideline has the benefit of substantially reducing the cost of guideline development, thus enabling this guideline to be developed in a timely manner. However, this process is limited by a reduced ability to assess new evidence published since the original guidelines were completed.

### Justifications summary

Recommendation 23(a) was excluded based on substantial differences in judgements to the IWGDF for the certainty of evidence and balance of effects (Table 2) and due to inclusion of a heterogeneous population that was considered too broad for the recommendation. In addition, Recommendation 21(a) was considered to already cover the subgroup of patients where evidence exists to support a shorter duration of therapy for diabetes-related foot osteomyelitis.

Recommendations 12, 16, 17 and 18 were adapted in large part due to differences in the population. In particular, there is no local evidence to suggest a need for a difference in empiric antibiotic management in patients in temperate or tropical locations, however, the chronicity of infection does impact the bacteria identified on culture.

### Implementation considerations summary

Beyond the implementation considerations identified for specific patient subgroups, key themes that were identified as relating to implementation included variable accessibility and variable expertise. Specific diagnostic techniques and treatment approaches identified as having variable availability across geographical locations and secondary and tertiary centres included procalcitonin, percutaneous bone biopsy, advanced imaging studies, restricted antibiotics, and surgical expertise. Furthermore, there is reduced expertise in the use of some diagnostic tests such as procalcitonin and percutaneous bone biopsy. While alternative options for procalcitonin such as CRP and ESR are widely available, the panel recommended that expertise in percutaneous bone biopsy be developed more widely.

The panel highlighted that choice of antibiotic regimen should include multiple considerations including a number that are patient-related. Considerations include likely or proven causative pathogen(s) and their antibiotic susceptibilities, expected efficacy, severity of infection, route of administration, adverse drug reactions, local antibiotic resistance patterns, appropriate antimicrobial stewardship, antibiotic restrictions, cost, access, likelihood of drug interactions, and patient preferences for route of administration and risk of adverse reactions.

### Subgroup considerations summary

#### Geographically remote people

People living in geographically remote areas face a number of barriers to effective implementation of this guideline. Barriers include reduced access to diagnostic services including basic services such as X-ray and advanced services such as MRI or PET scan, delays in time to results for biomarkers or pathological sampling and reduced access to surgical and specialist foot expertise. In addition, options for treatment may be impacted with hospitalisation unavailable locally and reduced local access to intravenous antibiotics or surgery.

An inability to undergo local treatment may impact the choice of treatment for some patients who wish to be treated in their local community, with an increased preference given to non-surgical interventions, outpatient parenteral therapy, oral antibiotics or use of telehealth by some individuals. In many circumstances, treatment will still be required, and it is important that remote centres have clear referral pathways (including criteria for referral and who to contact) to ensure access to timely advice and transfer mechanisms. In addition, the use of technology such as telehealth appointments and patient-centred co-management arrangements such as joint appointments with general practitioners and specialists should be considered where feasible.

#### Aboriginal and Torres Strait islander peoples

Given the increased risk of complications from diabetes-related foot infections, including amputations, in Aboriginal and Torres Strait Islander Peoples, it is vital that guidelines be adjusted to ensure inclusivity of this population. Cultural and language barriers need to be carefully assessed and mitigated through the support of Aboriginal health workers, Aboriginal liaison officers and interpreters as much as possible. Clinicians should aim to explore each patient’s understanding of their diabetes-related foot infection including predisposing factors, prognosis and potential treatment options.

As described in the section above, many of the potential barriers to implementation of the guideline that relate to geographically remote people also relate to a substantial proportion of Aboriginal and Torres Strait Islander Peoples due to 20% of Aboriginal and Torres Strait Islander Peoples living rurally [45] and a substantial proportion living in remote locations. An inability to undergo treatment locally may impact the choice of treatment for some patients who wish to be treated on country and/or near their local community, and clinicians should discuss alternative treatment options with patients including associated benefits and risks.

The panel highlighted that some Aboriginal and Torres Strait Islanders may wish to use a combination of traditional and western medicine and that clinicians should approach this with an open mind that positively fosters the therapeutic relationship and encourages engagement with medical services. In certain circumstances traditional medicine may be a potential harm, however, this should be addressed in a sensitive and culturally appropriate manner.

Prescribers should consider empiric MRSA coverage in patients known to be colonised with MRSA or those living in areas with a high prevalence of MRSA. An increased rate of MRSA has been identified in some Aboriginal and Torres Strait Islanders populations. For example, a study from Darwin found over 40% of Aboriginal and Torres Strait Islander patients with a diabetes-related foot infection had associated MRSA [5].

#### Other subgroup considerations

Prescribers should consider empiric MRSA coverage in patients known to be colonised with MRSA or those living in areas with a high prevalence of MRSA.

### Monitoring considerations summary

The panel recognises that different services will undertake this process differently, however, they noted that monitoring and evaluation forms a vital component of best-practice clinical management of diabetes-related foot infections and the principles followed should be similar for all services. The panel suggests that services undertake an audit of patient outcomes every 12 months at a minimum. To facilitate this, minimum data [46] should be collected on patients’ treatment approaches (including antibiotic and surgical management) and outcomes. Outcomes should be compared over time and to external units where possible.

### Future research considerations summary

The adaptation and development of this guideline highlights the low number of clinically relevant high quality studies that exist to assess the diagnostic and in particular the treatment options for diabetes-related foot infections. Of the 35 new sub-recommendations only two were rated as having a high quality of evidence and 12 as moderate quality of evidence. A need for future research was identified for the majority of the 35 guideline recommendations. In particular there is a need, where possible, for future research to follow strong methodological processes such as RCTs, with uniform patient-centred outcome measures.

Clinical researchers need to continue to evolve consensus definitions for the diagnosis, monitoring and resolution of both soft tissue and deep structure infections. There is a need for robust validation of assessment tools that focus on the specific clinical signs and symptoms of infection within the complex environment of diabetes-related foot disease. These should be accompanied by standardised microbiological sampling and processing as well as radiological reporting methods. Alongside this, the broader community must identify appropriate patient reported outcomes to ensure large studies can be designed that are pragmatic and relevant; comparing diagnostic techniques, antibiotic route, duration and type, surgical intervention and adjunctive therapies.

In addition, future research should consider clinically relevant questions. For example, although a number of randomised controlled trials have assessed antibiotic therapy for diabetes-related foot infections, most have been non-inferiority studies comparing a new antibiotic with standard therapy and few have directly compared commonly used antibiotics and thus have not impacted daily clinical practice.

## Conclusion

Adaptation of the 2019 IWGDF’s Infection Guidelines has enabled the development of 35 evidence-based diabetes-related foot infection recommendations to assist practitioners in secondary and tertiary settings in the Australian context from the multiple disciplines that diagnose and treat diabetes-related foot infections. In combination with simplified clinical pathway tools, they provide an evidence-based framework to ensure best management of individuals with diabetes-related foot infections across Australia and highlight considerations that are needed in specific patient subgroups, such as Aboriginal and Torres Strait Islander Peoples and geographically remote populations.

## Supplementary Information

**Additional file 1.**

## Data Availability

Data sharing is not applicable to this article as no datasets were generated or analysed during the current study.

## Abbreviations

CRP: C-reactive protein
CT: Computed tomography
ESR: Erythrocyte sedimentation ration
EtD: Evidence to Decision
GRADE: Grading of Recommendations Assessment, Development and Evaluation
IDSA: Infectious Diseases Society of America
IWGDF: International Working Group of the Diabetic Foot
MDT: Multidisciplinary team
MRSA: methicillin resistant *Staphylococcus aureus*
NHMRC: National Health and Medical Research Council
PICO: Population, intervention, control and outcomes
RCT: Randomised controlled trial
SIRS: Systemic inflammatory response syndrome
