# Supplementary material for: Australian guideline on management of diabetes-related foot infection: part of the 2021 Australian evidence-based guidelines for diabetes-related foot disease
Source: J Foot Ankle Res. 2022 Jun 9;15:47. doi: 10.1186/s13047-022-00545-4 (PMC9178854; doi:10.1186/s13047-022-00545-4)
Supplement: Supplementary file 1 — Additional file 1. [file 13047_2022_545_MOESM1_ESM.pdf]

Australian guideline on management of diabetes-related foot infection: Part of the 2021 Australian evidence-based guidelines for diabetes-related foot disease

Commons RJ, Charles J, Cheney J, Lynar SA, Malone M, Raby E, on behalf of the Australian Diabetes-related Foot Disease Guidelines & Pathways Project

Table of contents

	Page
**Table S1:** Detailed justifications for judgements for Recommendation 12	2
**Table S2:** Detailed justifications for judgements for Recommendation 16	4
**Table S3:** Detailed justifications for judgements for Recommendation 17	5
**Table S4:** Detailed justifications for judgements for Recommendation 18	6
**Table S5:** Detailed justifications for judgements for Recommendation 21a	7
**Table S6:** Detailed justifications for judgements for Recommendation 23a	8
**Table S7:** Detailed justifications for judgements for Recommendation 25b	10
**References S8:** Additional references from Supplementary material	11

Supplementary Table S1: Detailed justifications for judgements for Recommendation 12 according to GRADE EtD framework (17, 24, 25).

Item	Judgement	Rationale
Problem	Agreed	The panel agreed with the IWGDF that the current evidence supports that diabetes-related foot infections are a serious and urgent health problem, both internationally (10) and in Australia (5, 6).
Desirable effects	Unsure	The panel was unsure if they agreed with the IWGDF as it was unclear how substantial the IWGDF considered the undesirable effects to be for this recommendation. The panel felt that although there is no direct evidence informing this recommendation, there was evidence that parenteral antibiotics for severe (grade 4) infections would provide moderate desirable effects. This judgement considered that most published studies treat patients on an inpatient basis with intravenous antibiotics initially, severe (grade 4) infections can be life and limb-threatening, parenteral administration allows higher blood concentrations of antibiotics and most organisms are killed in a dose-dependent manner suggesting that early treatment with parenteral antibiotics will likely improve cure.
Undesirable effects	Unsure	The panel was unsure if they agreed with the IWGDF as it was unclear how substantial the IWGDF considered the undesirable effects to be for this recommendation. The panel considered the undesirable effects to be trivial given the moderate desirable effects on clinical cure, mortality and amputation identified for parenteral treatment for severe (grade 4) infections. In this context the inconvenience of parenteral treatment and likely admission was considered minor.
Quality (or certainty) of evidence	Disagreed	The panel disagreed with the IWGDF slightly, downgrading the quality of evidence rating from low to very low, although it was noted that the minimal evidence rating used in the IWGDF guidelines was low. This was based on a lack of direct evidence, but parenteral therapy for severe (grade 4) infections is considered standard of care according to expert opinion.
Values	Unsure	The IWGDF assessed a number of outcomes relevant to infection including clinical cure of infection, requirement for lower extremity amputation, occurrence of a new infection, death, hospitalisation, resolution of a foot ulcer, eradication of microbial pathogens, quality of life, adverse effects, or cost of treatment (47). However, no critical outcome was identified. As such the panel defined the critical outcome as clinical cure of infection based on a recent review of reporting standards by Jeffcoate et al (48). The panel noted that there was possible uncertainty in the degree to which patients would value cure of infection as compared with other outcomes such as amputation, ulcer healing and mortality.
Balance of effects	Agreed	The panel agreed with the IWGDF that moderately desirable effects outweighed the trivial undesirable effects and strongly favoured the use of parenteral antibiotics for severe (grade 4) infections.
Acceptability	Unsure	The panel was unsure if they agreed with the IWGDF as it was unclear as to what the IWGDF acceptability rating was for this recommendation. However, it was the judgement of the panel that the use of parenteral antibiotics for severe (grade 4) skin and soft tissue diabetes-related foot infections would be acceptable to most patients and providers in the Australian setting. This relates to the severity of the illness and potential substantial benefits from parenteral antibiotics compared with potentially less effective oral antibiotics.
Feasibility	Unsure	The panel was unsure if it agreed with the IWGDF as the IWGDF did not report on the feasibility or applicability of this recommendation. However, it was the panel’s judgement that the use of parenteral antibiotics for severe (grade 4) skin and soft tissue diabetes-related foot

	infections were feasible in the Australian setting. Given that this intervention is consistent with current practice it was considered sustainable. Furthermore, there were considered to be few barriers to implementation in most secondary and tertiary healthcare settings in Australia although it was noted that some remote locations may require initial intramuscular administration of antibiotics or once off intravenous antibiotics before transferring to a larger facility.

Supplementary Table S2: Detailed justifications for judgements for Recommendation 16 according to GRADE EtD framework (17, 24, 25).

Item	Judgement	Rationale
Problem	Agreed	The panel agreed with the IWGDF that the current evidence supports that diabetes-related foot infections are a serious and urgent health problem, both internationally (10) and in Australia (5, 6).
Desirable effects	Agreed	The panel was unsure if they agreed with the IWGDF as it was unclear how substantial the IWGDF considered the desirable effects to be for this recommendation. Although there are likely trivial benefits to clinical cure and other infection related outcomes from using narrower spectrum antibiotics, the panel felt that there were small desirable effects given that the use of narrower spectrum antibiotics where efficacy is otherwise equivalent would likely reduce the risk of developing antibiotic resistance and may reduce the risk of some adverse events.
Undesirable effects	Unsure	The panel was unsure if they agreed with the IWGDF as it was unclear how substantial the IWGDF considered the undesirable effects to be for this recommendation. The panel considered the undesirable effects to be trivial given that for patients who have not recently received antibiotic therapy and have an acute mild (grade 2) infection descriptive studies of microbiology describe predominantly Gram-positive aerobic organisms (10, 35, 47). Furthermore, treatment of solely mild (grade 2) infection allows rapid escalation should clinical deterioration occur.
Quality (or certainty) of evidence	Agreed	The panel agreed with the IWGDF that the quality of evidence rating was low, based on a lack of evidence comparing patient outcomes between narrower and broader spectrum antibiotics.
Values	Unsure	The IWGDF assessed a number of outcomes relevant to infection including clinical cure of infection, requirement for lower extremity amputation, occurrence of a new infection, death, hospitalisation, resolution of a foot ulcer, eradication of microbial pathogens, quality of life, adverse effects, or cost of treatment (47). However, no critical outcome was identified. As such the panel defined the critical outcome as clinical cure of infection based on a recent review of reporting standards by Jeffcoate et al (48). The panel noted that there was possible uncertainty in the degree to which patients would value cure of infection as compared with other outcomes such as amputation, ulcer healing and mortality.
Balance of effects	Agreed	The panel agreed with the IWGDF that the small desirable effects outweighed the trivial undesirable effects and weakly favoured the use of antibiotics targeting aerobic Gram-positive pathogens in cases of a mild (grade 2) acute diabetes-related foot infection where patients have not recently received antibiotic therapy.
Acceptability	Unsure	The panel was unsure if they agreed with the IWGDF as it was unclear what the IWGDF acceptability rating was for this recommendation. However, it was the judgement of the panel that this recommendation would likely be acceptable in the Australian context. The panel noted that there were no identified differences in microbiology in mild (grade 2) acute infections in more tropical and more temperate regions of Australia. Furthermore, they noted that local studies have demonstrated chronic infections to be more commonly polymicrobial (35) and this is reflected in local antibiotic treatment guidelines (33).
Feasibility	Unsure	The panel was unsure if it agreed with the IWGDF as the IWGDF did not report on the feasibility or applicability of this recommendation. However, it was the panel's judgement that the use of narrower spectrum antibiotics for mild (grade 2) acute infections was feasible in the Australian setting, both in more tropical and more temperate regions.

Supplementary Table S3: Detailed justifications for judgements for Recommendation 17 according to GRADE EtD framework (17, 24, 25).

Item	Judgement	Rationale
Problem	Agreed	The panel agreed with the IWGDF that the current evidence supports that diabetes-related foot infections are a serious and urgent health problem, both internationally (10) and in Australia (5, 6).
Desirable effects	Agreed	The panel was unsure if they agreed with the IWGDF as it was unclear how substantial the IWGDF considered the desirable effects to be for this recommendation. The panel felt there were likely moderate benefits to clinical cure and other infection related outcomes from using broader spectrum antibiotics in the subgroups of patients specified and these groups are more likely to have polymicrobial and anaerobic infections and the risk of not treating these organisms may worsen infection outcomes.
Undesirable effects	Unsure	The panel was unsure if they agreed with the IWGDF as it was unclear how substantial the IWGDF considered the undesirable effects to be for this recommendation. The panel considered the undesirable effects to be trivial given the potential desirable effects on clinical cure and other infection outcomes compared with a decreased risk of developing antibiotic resistance.
Quality (or certainty) of evidence	Agreed	The panel agreed with the IWGDF that the quality of evidence rating was low, based on a lack of evidence comparing patient outcomes between narrower and broader spectrum antibiotics.
Values	Unsure	The IWGDF assessed a number of outcomes relevant to infection including clinical cure of infection, requirement for lower extremity amputation, occurrence of a new infection, death, hospitalisation, resolution of a foot ulcer, eradication of microbial pathogens, quality of life, adverse effects, or cost of treatment (47). However, no critical outcome was identified. As such the panel defined the critical outcome as clinical cure of infection based on a recent review of reporting standards by Jeffcoate et al (48). The panel noted that there was possible uncertainty in the degree to which patients would value cure of infection as compared with other outcomes such as amputation, ulcer healing and mortality.
Balance of effects	Agreed	The panel agreed with the IWGDF that the small desirable effects outweighed the trivial undesirable effects and weakly favoured the use of antibiotics targeting aerobic Gram-positive pathogens in cases of a mild (grade 2) acute diabetes-related foot infection where patients have not recently received antibiotic therapy.
Acceptability	Unsure	The panel was unsure if they agreed with the IWGDF as it was unclear as to what the IWGDF acceptability rating was for this recommendation. However, it was the judgement of the panel that this recommendation would likely be acceptable in the Australian context. Consistent with the judgements of the panel in Recommendation 16, the panel noted that there were no identified differences in microbiology in mild (grade 2) infections in more tropical and more temperate regions of Australia. Furthermore, they noted that local studies have demonstrated chronic infections to be more commonly polymicrobial (35) and this is reflected in local antibiotic treatment guidelines (33).
Feasibility	Unsure	The panel was unsure if it agreed with the IWGDF as the IWGDF did not report on the feasibility or applicability of this recommendation. However, it was the panel’s judgement that the use of broader spectrum antibiotics for chronic infections was feasible in the Australian setting, both in more tropical and more temperate regions.

Supplementary Table S4: Detailed justifications for judgements for Recommendation 18 according to GRADE EtD framework (17, 24, 25).

Item	Judgement	Rationale
Problem	Agreed	The panel agreed with the IWGDF that the current evidence supports that diabetes-related foot infections are a serious and urgent health problem, both internationally (10) and in Australia (5, 6).
Desirable effects	Agreed	The panel was unsure if they agreed with the IWGDF as it was unclear how substantial the IWGDF considered the desirable effects to be for this recommendation. The panel felt there were likely small benefits to clinical cure and other infection related outcomes from using empiric *P. aeruginosa* coverage in the subgroups of patients specified as these groups were more likely to have pseudomonal infection and the risk of not treating this may worsen infection outcomes.
Undesirable effects	Unsure	The panel was unsure if they agreed with the IWGDF as it was unclear how substantial the IWGDF considered the undesirable effects to be for this recommendation. Similar to Recommendation 17, the panel considered the undesirable effects to be trivial given the potential desirable effects on clinical cure and other infection outcomes compared with a decreased risk of developing antibiotic resistance.
Quality (or certainty) of evidence	Agreed	The panel agreed with the IWGDF that the quality of evidence rating was low, based on a lack of evidence comparing patient outcomes between patients treated with and without empiric *P. aeruginosa* coverage.
Values	Unsure	The IWGDF assessed a number of outcomes relevant to infection including clinical cure of infection, requirement for lower extremity amputation, occurrence of a new infection, death, hospitalisation, resolution of a foot ulcer, eradication of microbial pathogens, quality of life, adverse effects, or cost of treatment (47). However, no critical outcome was identified. As such the panel defined the critical outcome as clinical cure of infection based on a recent review of reporting standards by Jeffcoate et al (48). The panel noted that there was possible uncertainty in the degree to which patients would value cure of infection as compared with other outcomes such as amputation, ulcer healing and mortality.
Balance of effects	Agreed	The panel agreed with the IWGDF that the small desirable effects outweighed the trivial undesirable effects and weakly favoured the use of empiric *P. aeruginosa* coverage in the subgroups of patients specified.
Acceptability	Unsure	The panel was unsure if they agreed with the IWGDF as it was unclear what the IWGDF acceptability rating was for this recommendation. However, it was the judgement of the panel that this recommendation would likely be acceptable in the Australian context. The panel noted that differences in identification of *P. aeruginosa* from diabetes-related foot infections in temperate and tropical Australia were not well described and that *P. aeruginosa* could occur in temperate regions; being present in 22% of chronic infections in a study from Melbourne, Australia (35).
Feasibility	Unsure	The panel was unsure if it agreed with the IWGDF as the IWGDF did not report on the feasibility or applicability of this recommendation. However, it was the panel's judgement that the use of empiric *P. aeruginosa* coverage in specific patient subgroups was feasible in the Australian setting.

Supplementary Table S5: Detailed justifications for judgements for Recommendation 21a according to GRADE EtD framework (17, 24, 25).

Item	Judgement	Rationale
Problem	Agreed	The panel agreed with the IWGDF that the current evidence supports that diabetes-related foot infections are a serious and urgent health problem, both internationally (10) and in Australia (5, 6).
Desirable effects	Agreed	The panel agreed with the IWGDF that the desirable effects of antibiotic versus surgical management were substantial. The panel felt that although there was no clear benefit one way or the other on clinical cure for antibiotic management for uncomplicated forefoot osteomyelitis over surgical management (37), there were likely moderate benefits through avoiding amputation, adverse events and hospitalisation.
Undesirable effects	Agreed	The panel agreed with the IWGDF that the undesirable effects of antibiotic versus surgical management were minimal. The panel considered the undesirable effects to be trivial given the minimal difference in clinical cure with antibiotic management or surgical management (37) and the potential undesirable effects of surgery such as amputation, foot deformity and hospitalisation.
Quality (or certainty) of evidence	Agreed	The panel agreed with the IWGDF that the quality of evidence rating was moderate, based on one RCT and two retrospective cohort studies (47).
Values	Unsure	The IWGDF assessed a number of outcomes relevant to infection including clinical cure of infection, requirement for lower extremity amputation, occurrence of a new infection, death, hospitalisation, resolution of a foot ulcer, eradication of microbial pathogens, quality of life, adverse effects, or cost of treatment (47). However, no critical outcome was identified. As such the panel defined the critical outcome as clinical cure of infection based on a recent review of reporting standards by Jeffcoate et al (48). The panel noted that there was possible uncertainty in the degree to which patients would value cure of infection as compared with other outcomes such as amputation, ulcer healing and mortality.
Balance of effects	Agreed	The panel agreed with the IWGDF that the moderate desirable effects outweighed the trivial undesirable effects and strongly favoured the use of antibiotics without surgical resection in uncomplicated forefoot osteomyelitis.
Acceptability	Unsure	The panel was unsure if they agreed with the IWGDF as it was unclear what the IWGDF acceptability rating was for this recommendation. However, it was the judgement of the panel that antibiotic therapy without surgery would likely be acceptable to most patients and providers in the Australian context and would be preferred by many patients due to the ability to avoid surgery.
Feasibility	Unsure	The panel was unsure if it agreed with the IWGDF as the IWGDF did not report on the feasibility or applicability of this recommendation. However, it was the panel's judgement that the use of antibiotics for uncomplicated forefoot osteomyelitis was feasible in the Australian setting; likely more broadly than surgical intervention although there was still a need for surgical expertise to identify patients that need surgery.

Supplementary Table S6: Detailed justifications for judgements for Recommendation 23a according to GRADE EtD framework (17, 24, 25).

Item	Judgement	Rationale
Problem	Agreed	The panel agreed with the IWGDF that the current evidence supports that diabetes-related foot infections are a serious and urgent health problem, both internationally (10) and in Australia (5, 6).
Desirable effects	Unsure	The panel was unsure if they agreed with the IWGDF as it was unclear how substantial the IWGDF considered the undesirable effects to be for this recommendation. The panel felt that the benefit of 6 weeks or less of antibiotics over a longer duration of antibiotics was trivial in the heterogeneous group of patients with osteomyelitis, with no likely improvement in clinical cure, amputation risk, or ulcer healing but some benefits in reduced adverse reactions, cost and development of antibiotic resistance. Although cure of infection may occur in some patients including acute uncomplicated osteomyelitis, most of these patients were described in other recommendations such as Recommendation 21a and Recommendation 25b.
Undesirable effects	Disagreed	The panel disagreed with the IWGDF as the undesirable effects of a 6 week or less course of antibiotics compared with a longer course of antibiotics were considered to vary depending on the subtype of osteomyelitis being treated. While acute uncomplicated or minimal residual post-surgical osteomyelitis was considered more likely to have clinical cure of infection with a shorter antibiotic course, chronic osteomyelitis or complicated minimally-resected osteomyelitis were likely to require a more prolonged course. The panel also noted that there were circumstances where patient and provider preferences may mean that lifelong antibiotics were reasonable (i.e. to avoid amputation).
Quality (or certainty) of evidence	Disagreed	Given the breadth of clinical presentations of diabetes-related foot osteomyelitis are not well represented in the available literature and in many cases rely on small studies of subgroups of patients the panel considered the certainty of evidence to be low.
Values	Unsure	The IWGDF assessed a number of outcomes relevant to infection including clinical cure of infection, requirement for lower extremity amputation, occurrence of a new infection, death, hospitalisation, resolution of a foot ulcer, eradication of microbial pathogens, quality of life, adverse effects, or cost of treatment (47). However, no critical outcome was identified. As such the panel defined the critical outcome as clinical cure of infection based on a recent review of reporting standards by Jeffcoate et al (48). The panel noted that there was possible uncertainty in the degree to which patients would value cure of infection as compared with other outcomes such as amputation, ulcer healing and mortality.
Balance of effects	Disagreed	The panel disagreed with the IWGDF due to variation in desirable and undesirable effects relating to the different subgroups of patients with osteomyelitis and different patient considerations. For example, in a patient with uncomplicated acute osteomyelitis antibiotics for 6 weeks or less is likely favoured. However, in a patient with chronic calcaneal osteomyelitis that is adamant they do not want an amputation, long term suppressive antibiotics may be considered favourable.
Acceptability	Unsure	The panel was unsure if they agreed with the IWGDF as it was unclear what the IWGDF acceptability rating was for this recommendation. In addition, due to the heterogenous nature of osteomyelitis the panel noted that acceptability was likely to vary. For example, in a hypothetical case of diabetes-related foot osteomyelitis managed with limited debridement to avoid amputation, a survey of Australian specialists reported wide variation in practice with many clinicians

		likely to recommend much longer than 6 weeks of therapy (49). Historically, chronic osteomyelitis has been treated for longer duration and this is reflected in the Australian infectious diseases' guidelines. However, the panel noted that recent publications in other areas of infectious diseases have identified several disease processes where shorter courses of antibiotics are favoured (50-52) and this fits with a broader acceptance and promotion of antimicrobial stewardship principles.
Feasibility	Unsure	The panel was unsure if it agreed with the IWGDF as the IWGDF did not report on the feasibility or applicability of this recommendation. However, it was the panel's judgement that the recommendation was probably feasible in the Australian setting, should the recommendation be considered appropriate. The panel noted that some aspects of the recommendation were less feasible than others. For example, relatively few centres have adequate expertise to perform diagnostic (percutaneous) bone biopsy, although it is recognised that it would be ideal if this was available more widely. In addition, 'clinical improvement' of osteomyelitis is not clearly defined and there are no standardised clinical or research assessment tools available to guide this assessment. A 2 to 4 week duration was noted to be a relatively short time to assess changes on plain imaging or bloods unless there was marked progression associated with more acute presentations.

Supplementary Table S7: Detailed justifications for judgements for Recommendation 25b according to GRADE EtD framework (17, 24, 25).

Item	Judgement	Rationale
Problem	Agreed	The panel agreed with the IWGDF that the current evidence supports that diabetes-related foot infections are a serious and urgent health problem, both internationally (10) and in Australia (5, 6).
Desirable effects	Agreed	The panel agreed with the IWGDF that there were likely desirable effects from antibiotic treatment based on the results of post-resection bone biopsy compared to no treatment. The panel felt that there were small desirable benefits given that identifying and treating unexpected residual infection would likely lead to improved clinical cure of infection and reduced risk of relapse.
Undesirable effects	Agreed	The panel agreed with the IWGDF that there were likely minimal undesirable effects from antibiotic treatment based on the results of post-resection bone biopsy compared to no treatment. The panel considered the undesirable effects to be trivial and to relate to a risk of false positive biopsy, which as identified by the IWGDF can result from contamination of samples. This risk can be reduced by careful aseptic technique, use of new sterile instruments to conduct the biopsy and combined microbiological and histological assessment. If osteomyelitis was falsely diagnosed patients would potentially undergo prolonged antibiotic therapy and the associated risks of adverse drug reactions, line-related complications, hospitalisation, and travel.
Quality (or certainty) of evidence	Agreed	The panel agreed with the IWGDF that the quality of evidence rating was moderate, based on three studies which found that patients were more likely to have poor outcomes if they had biopsy evidence of osteomyelitis after resection compared with negative biopsy results (10).
Values	Unsure	The IWGDF assessed a number of outcomes relevant to infection including clinical cure of infection, requirement for lower extremity amputation, occurrence of a new infection, death, hospitalisation, resolution of a foot ulcer, eradication of microbial pathogens, quality of life, adverse effects, or cost of treatment (47). However, no critical outcome was identified. As such the panel defined the critical outcome as clinical cure of infection based on a recent review of reporting standards by Jeffcoate et al (48). The panel noted that there was possible uncertainty in the degree to which patients would value cure of infection as compared with other outcomes such as amputation, ulcer healing and mortality.
Balance of effects	Agreed	The panel agreed with the IWGDF that the small desirable effects outweighed the trivial undesirable effects and strongly favoured the use of antibiotics if post-resection specimens were consistent with osteomyelitis.
Acceptability	Unsure	The panel was unsure if they agreed with the IWGDF as it was unclear what the IWGDF acceptability rating was for this recommendation. However, it was the judgement of the panel that the use of antibiotics, if post-resection specimens were consistent with osteomyelitis, would likely be acceptable to most patient and providers in the Australian context and would be preferred by many patients over clinical failure.
Feasibility	Unsure	The panel was unsure if it agreed with the IWGDF as the IWGDF did not report on the feasibility or applicability of this recommendation. However, it was the panel's judgement that the recommendation was feasible in the Australian setting, although it was noted that histopathology may have a slower turnaround time which may vary by location and have substantial cost differences.

Supplementary References S8: Additional references from Supplementary material

47. Peters EJG, Lipsky BA, Senneville E, Abbas ZG, Aragon-Sanchez J, Diggle M, et al. Interventions in the management of infection in the foot in diabetes: a systematic review. Diabetes Metab Res Rev. 2020;36 Suppl 1:e3282.

48. Jeffcoate WJ, Bus SA, Game FL, Hinchliffe RJ, Price PE, Schaper NC, et al. Reporting standards of studies and papers on the prevention and management of foot ulcers in diabetes: required details and markers of good quality. Lancet Diabetes Endocrinol. 2016;4(9):781-8.

49. Commons RJ, Raby E, Athan E, Bhally H, Chen S, Guy S, et al. Managing diabetic foot infections: a survey of Australasian infectious diseases clinicians. J Foot Ankle Res. 2018;11:13.

50. Dawson-Hahn EE, Mickan S, Onakpoya I, Roberts N, Kronman M, Butler CC, et al. Short-course versus long-course oral antibiotic treatment for infections treated in outpatient settings: a review of systematic reviews. Fam Pract. 2017;34(5):511-9.

51. Royer S, DeMerle KM, Dickson RP, Prescott HC. Shorter Versus Longer Courses of Antibiotics for Infection in Hospitalized Patients: A Systematic Review and Meta-Analysis. J Hosp Med. 2018;13(5):336-42.

52. Tansarli GS, Andreatos N, Pliakos EE, Mylonakis E. A Systematic Review and Meta-analysis of Antibiotic Treatment Duration for Bacteremia Due to Enterobacteriaceae. Antimicrob Agents Chemother. 2019;63(5).
